# Enalapril and Enalaprilat Pharmacokinetics in Children with Heart Failure Due to Dilated Cardiomyopathy and Congestive Heart Failure after Administration of an Orodispersible Enalapril Minitablet (LENA-Studies)

**DOI:** 10.3390/pharmaceutics14061163

**Published:** 2022-05-30

**Authors:** Stephanie Laeer, Willi Cawello, Bjoern B. Burckhardt, László Ablonczy, Milica Bajcetic, Johannes M. P. J. Breur, Michiel Dalinghaus, Christoph Male, Saskia N. de Wildt, Jörg Breitkreutz, Muhammed Faisal, Anne Keatley-Clarke, Ingrid Klingmann, Florian B. Lagler

**Affiliations:** 1 Institute of Clinical Pharmacy and Pharmacotherapy, Heinrich-Heine-Universitaet Düsseldorf, 40225 Duesseldorf, Germany; cawello@uni-duesseldorf.de (W.C.); bjoern.burckhardt@hhu.de (B.B.B.); mufai100@hhu.de (M.F.); 2 Goettsegen György Hungarian Institute of Cardiology (HPHC), 1450 Budapest, Hungary; ablonczyl@gmail.com; 3 Univerzitetska Dečja Klinika (UDK), University Children Hospital, School of Medicine, University of Belgrade, 11129 Belgrade, Serbia; mbajcetic@doctor.com; 4 University Medical Center Utrecht, Wilhelmina Children’s Hospital, 3584 CX Utrecht, The Netherlands; h.breur@umcutrecht.nl; 5 Division of Pediatric Cardiology, Erasmus MC Sophia Children’s Hospital, 3000 CA Rotterdam, The Netherlands; m.dalinghaus@erasmusmc.nl; 6 Department of Paediatrics and Adolescent Medicine, Medical University of Vienna, 1090 Vienna, Austria; christoph.male@meduniwien.ac.at; 7 Intensive Care and Department of Pediatric Surgery, Erasmus MC Sophia Children’s Hospital, 3015 GJ Rotterdam, The Netherlands; saskia.dewildt@radboudumc.nl; 8 Department of Pharmacology and Toxicology, Radboud Institute of Health Sciences, Radboud University Medical Center, 6500 HB Nijmegen, The Netherlands; 9 Ethicare GmbH, 45721 Haltern am See, Germany; joerg.breitkreutz@hhu.de; 10 The Children’s Heart Federation, London EC2A 3NW, UK; akc@chfed.org.uk; 11 Pharmaplex Bvba, B-1970 Wezembeek-Oppem, Belgium; iklingmann@pharmaplex.be; 12 Paracelsus Medizinische Privatuniversität, 5020 Salzburg, Austria; f.lagler@crcs.at

**Keywords:** pediatric cardiology, heart failure, dilated cardiomyopathy, congenital heart disease, ACEIs, enalapril, orodispersible minitablets

## Abstract

Angiotensin-converting enzyme inhibitors (ACEI), such as enalapril, are a cornerstone of treatment for pediatric heart failure which is still used off-label. Using a novel age-appropriate formulation of enalapril orodispersible minitablets (ODMTs), phase II/III open-label, multicenter pharmacokinetic (PK) bridging studies were performed in pediatric patients with heart failure due to dilated cardiomyopathy (DCM) and congenital heart disease (CHD) in five participating European countries. Children were treated for 8 weeks with ODMTs according to an age-appropriate dosing schedule. The primary objective was to describe PK parameters (area under the curve (AUC), maximal concentration (Cmax), time to reach maximal concentration (t-max)) of enalapril and its active metabolite enalaprilat. Of 102 patients, 89 patients (*n* = 26, DCM; *n* = 63 CHD) were included in the primary PK endpoint analysis. Rate and extent of enalapril and its active metabolite enalaprilat were described and etiology and age could be identified as potential PK modifying factors. The dosing schedule appeared to be tolerated well and did not result in any significant drug-related serious adverse events. The PK analysis and the lack of severe safety events supports the applied age-appropriate dosing schedule for the enalapril ODMTs.

## 1. Introduction

Enalapril is an ACE inhibitor (ACEI) commonly administered off-label to young children with heart failure by using extemporaneous formulations. Enalapril is not labelled for patients younger than 6 years of age (<20 kg) in the EU [1]. Enalapril’s beneficial effects, such as improving cardiac output and preventing cardiac hypertrophy and remodeling, are assumed similar to those in adults with heart failure due to cardiomyopathy or coronary heart disease [2,3]. The European Medicine Agency Expert Group Meeting on Paediatric Heart Failure considered enalapril as the first-line treatment for chronic heart failure in children [4]. Pediatric pharmacokinetic (PK), pharmacodynamic, and safety data for young children with heart failure are sparse and not at all described for any specific formulation [5,6,7].

Thus, the safe and effective use of enalapril in pediatric patients requires a detailed evaluation of the pharmacokinetics of enalapril and its active metabolite enalaprilat in pediatric heart failure following application of a novel oral formulation [8] because maturation and growth might affect absorption, distribution, metabolism, and elimination in the pediatric population. Developmental changes in drug absorption of orally administered drugs are well known [9]. The maturating expression levels of binding proteins [10] may influence the drug and metabolite tissue distribution [11]. The maturating metabolizing carboxylesterases enzyme expressions in the liver [12] responsible for the conversion of enalapril to enalaprilat [13], as well as the rapidly maturating glomerular filtration rate [14], may influence the renal elimination of enalapril and enalaprilat, especially in young children [15]. All these developmental changes may affect the systemic exposure of enalapril and enalaprilat and may support a pediatric dosing regimen.

Additionally, the pathophysiologic condition of heart failure may affect pharmacokinetic parameters. It is known from adults that systemic arterial hypoperfusion, neurohormonal activation, and venous congestion alter the [16] absorption [17], distribution [18], and renal and metabolic elimination [14] of drugs. In addition, a volume overload condition may cause pleural effusion, ascites, and anasarca and may alter the distribution of water-soluble drugs in these modified compartments [16]. For adult patients with heart failure and reduced cardiac output, enalaprilat exposure was significantly higher than in healthy adults and reduced volumes of distribution, clearance, and elimination were considered responsible for this [19,20].

Clinical presentations in pediatric heart failure, such as edema, growth failure, and circulatory derangements [21], may also alter the pharmacokinetics of drugs and may require dosing adjustments [18,22]. These pharmacokinetic changes may be indicated by pharmacokinetic parameters, such as AUC, Cmax, tmax, and clearance, which can be useful for determining pathophysiological effects on drug elimination and in adjusting the dose of drugs for safe and effective use.

As part of the LENA project, two novel formulations of orodispersible minitablets (ODMTs) [23] were developed containing 0.25 or 1.00 mg enalapril maleate in small-sized, directly compressed tablets (2 mm in diameter and height), which rapidly dissolve upon contact with water or saliva, suitable for pediatric administration. [8] Subsequently, a bioequivalence study in healthy adults was performed, showing comparable relative bioavailability and pharmacokinetics of the 10 ODMTs with 1.00 mg enalapril and an authorized marketed tablet formulation (Renitec 10 mg by MSD) [24,25].

Two clinical phase II/III prospective, open-label, multicenter PK bridging studies in children (1 month to younger than 12 years of age) with heart failure due to dilated cardiomyopathy (DCM) and congenital heart disease (CHD) and 1 common follow-up safety study for both populations were performed. Study patients were treated with the newly developed enalapril ODMTs based on an age-appropriate dosing regimen developed by physiologically based modelling and simulation. Patients were systematically assessed for PK, PD [26,27,28] clinical parameters and ODMT acceptability and palatability [8]. This manuscript describes the primary and secondary objective concerning the pharmacokinetic study’s endpoints, in which pediatric PK data of enalapril and enalaprilat were obtained in these children to characterize the rate and extent of the exposure in the pediatric population with DCM or CHD.

## 2. Materials and Methods

### 2.1. Study Design

The study was designed to determine the pharmacokinetics, pharmacodynamics, and safety of enalapril ODMTs in the pediatric population with heart failure. The data are from two multicenter phase II/III prospective, open-label, single, and multiple dose pharmacokinetic bridging studies with exploratory pharmacodynamic assessments. ACEI-pre-treated and ACEI-naïve children with heart failure due to DCM or CHD were enrolled in eight investigative sites and treated for 8 weeks, with the maximum tolerated dose following a defined dose titration scheme. Children with heart failure due to CHD were included from day 1 of age to younger than 6 years of age. Children with heart failure due to DCM were included from 1 month of age to younger than 12 years of age. The following reasons were the basis for the lower inclusion age in children with congenital heart disease. Patients with congenital heart disease such as ventricular septal defect are usually presented to hospital already in the neonatal age. Then, ACE inhibitor treatment will be started immediately to reduce symptoms of heart failure to gain time until children had achieved sufficient weight for surgical correction. In contrast, dilated cardiomyopathy in children appear symptomatic usually within the first three months of age. As the clinicians of the included clinical centers did not start ACE inhibitor treatment before first month of age for safety reasons, inclusion criterion for age was from 1 month of age for DCM patients. This had been also accepted by the regulatory authorities. For both etiologies of heart failure, a mean body weight of 2.5 kg was chosen as the lower limit for inclusion into the studies. The ethical committees at each participating institution approved the study protocols. Informed parental consent was obtained before each participant was enrolled in the study. Assent of participating children was obtained according to local IRB practices. The investigative sites were Austria, Germany, Hungary, The Netherlands (2 sites), Serbia (2 sites), and UK (EudraCT 2015-002335-17; EudraCT 2015-002396-18).

#### Study Outcomes

Details of the study design are described elsewhere [8]. The primary outcome of the pharmacokinetic bridging studies was the bioavailability of enalapril and enalaprilat in children with heart failure (area under the curve (AUC) within a dosing interval of 12 h, Cmax and Tmax), a descriptive PK investigation. A secondary objective analysis of the primary outcome by age strata was the investigation of the bioavailability of enalapril and enalaprilat by age subsets of 1 month to younger than 12 months, 12 months to younger than 6 years, and 6 years to younger than 12 years of the pediatric population with the same pharmacokinetic parameters.

Secondary outcomes were the RAAS markers as an exploratory PD investigation, brain natriuretic peptides (NT-proBNP), acceptability and palatability of the novel formulation, safety parameters including blood pressure and renal function, echocardiography (SF), rehospitalization due to heart failure including the need for heart transplantation or the institution of mechanical circulatory support, death due to worsening of the underlying disease, PD, and efficacy endpoint analyses to differentiate high and low output disease. Additionally, a pharmacogenomics and metabolomics sub-study was performed (see also in detail [8]).

### 2.2. Patient Selection

Patients were required to fulfill the following inclusion criteria. Diagnosis of heart failure due to CHD requiring after load reduction by drug therapy or diagnosis of DCM presenting with LV end-diastolic dimension > P95 and/or LV shortening fraction <25% in patients, resulting from different types of underlying cardiac disease with signs of decreased systolic LV function, and without ACEI treatment. Patients with ACEI pretreatment had to have documented evidence of having fulfilled these criteria before the start of ACEI therapy. Male and female patients aged 1 month to younger than 12 years for patients with DCM and from birth to younger than 6 years for patients with CHD and weight greater than 2.5 kg could be enrolled. Subjects could be naïve to ACEIs or already on ACEIs and willing to switch to enalapril ODMTs.

Patients were excluded if they had severe heart failure and/or end-stage heart failure precluding introduction or continuation of ACEI; too low blood pressure (e.g., <P5) for age; restrictive and hypertrophic cardiomyopathies; valvular obstruction (peak echocardiographic gradient more than 30 mm Hg); uncorrected severe peripheral stenosis of large arteries, including severe aortic coarctation; severe renal impairment with serum creatinine of more than two times the upper limit of normal according to the hospital’s test methodology; history of angioedema; and hypersensitivity to ACEI. The following concomitant medications were not permitted: dual ACEI therapy, renin inhibitors; angiotensin II antagonists; NSAIDs, except acetylsalicylic acid only for antiplatelet therapy; and any drug that might interfere with the absorption of enalapril. Patients were excluded if they were already enrolled in an interventional trial with an investigational drug, unless no interference with the current study could be shown.

Children were stratified into three groups by age: Group I, 1 day to younger than 12 months; Group II, 12 months to younger than 6 years; and Group III, children with heart failure due to cardiomyopathy of 6 years to less than 12 years of age.

### 2.3. Treatment

The study’s investigational medicinal products were orodispersible minitablets (ODMTs, Ethicare GmbH, Haltern am See, Germany) containing 2 different dose strengths of enalapril maleate: enalapril maleate 1.00 mg and enalapril maleate 0.25 mg. The ODMT was packed in tamper-evident high-density polyethylene bottles with a high-density polyethylene screw cap with desiccant. Each bottle contained 200 ODMTs of the same batch. The ODMTs were produced in accordance with good manufacturing practice by the pharmaceutical contract manufacturing company Pharbil Waltrop GmbH, member of the NextPharma group.

To optimize dosing reliability during the whole study and particularly during the titration period, an individual dose preparation process was implemented. The preparation of individual doses was performed at the clinical site or in the hospital pharmacy according to the local national requirements. The sites were provided with preprepared paper boxes that contained 18 holes for morning and evening doses in a 14-day treatment period plus reserve, empty bottles, and respective labels for the bottles and the box. The site was instructed to prepare the daily medication for the next treatment period according to the investigator’s prescription by counting the required dose strength and number of minitablets from the respective 200 ODMT bottles, filling them into the empty bottles, and labelling the bottles and the box. In addition, the site staff were requested to instruct the parents in the use of the medication in the box and to encourage the parents to bring the used boxes back at each visit. Ethicare GmbH supplied the empty containers, which were of the same quality as the ODMTs, thus ensuring drug stability. The bottle cap contained a desiccant to ensure low moisture for the individually prepared doses.

In case of very low doses (<0.25 mg), a protocol was developed that enabled the preparation of enalapril dispersions made from a single 0.25 mg ODMT within an oral syringe. The ODMT quickly disintegrated in exactly 10 mL of tap water drawn into the syringe [29]. After discarding a predefined (5 or 9 mL) volume, the desired volume of 1 or 5 mL containing the child-appropriate dose (0.025 or 0.125 mg, respectively) could be directly administered to the child’s mouth without further losses. This procedure had been confirmed to provide precise doses according to the specifications of European Pharmacopoeia’s monograph 2.9.6.

Administration of enalapril ODMT dispersions via nasogastric tubes had been demonstrated to be feasible for tubes made from polyvinyl chloride, polyurethane, or silicon and of different diameters (Charrière 5 to 10). The dispersion did not block the tubes, and the recovery rate was high (>99%) after a maximum of three rinses with water [29].

#### 2.3.1. Administration and Food Restrictions

The ODMT was administered to the patient by placing it in the patient’s cheek pouch. A drink of the patient’s/parent’s choice (e.g., breast milk, formula milk, cow milk, or water) was offered to facilitate swallowing, thus further minimizing the risk of choking. In case of administration per nasogastric tube, the ODMTs were dispersed in water just before administration. Patients did not need to follow a trial-specific diet or other food restrictions. However, parents were instructed not to mix the ODMTs into food. After medication administration, the parents were instructed to avoid any meal for 2 h.

#### 2.3.2. Method of Assigning Patients to Treatment Groups

All patients were allocated to treatment with enalapril ODMTs and to the study group according to their pretreatment. Patients with ACEI pretreatment were allocated to one group, and all ACEI-naive patients were allocated to another group. There was no randomization of patients in this trial, as there were no comparisons to other treatments or no treatment. All patients received the ODMT by individually adapted titration according to the individually defined dosing scheme. Once the optimal dose was reached, the patients received this dose until the end of the 8-week study period, with at least once or twice weekly visits.

#### 2.3.3. Selection of Doses in the Study

The study’s dosing regimen was determined using modelling and simulation, that is, physiologically based pharmacokinetic simulation (PBPK, SIMCYP^®^ v13.2) with the aim of achieving a similar exposure in the various pediatric age subsets to that achieved in adults. A similar approach as that described by Khalil [30,31] was used to develop the enalapril/enalaprilat pediatric PBPK model. Data from the literature, including adult pharmacokinetic data, physiological data, in vitro and preclinical data, and enalapril/enalaprilat specific information and physicochemical properties, such as molecular weight, lipophilicity, clearance, solubility, and intestinal permeability, were collated [32,33,34,35,36,37,38,39,40,41]. The commercially available modelling software package (SIMCYP^®^ v13.2) was used to provide the general model structure with a standard database of anatomical and physiological parameters for adults and children. Enalapril and enalaprilat specific data were incorporated to build first a coupled adult PBPK model for the prodrug and its metabolite before scaling it to children. A full PBPK model was used with enalapril, and a minimal PBPK model was used with enalaprilat (input Vss = 2.4 L/kg, Vsac = 0.4 L/kg, Clin/Clout = 13/0.1 L/h). The Advanced Distribution and Absorption Model (ADAM) was used for the absorption of enalapril. The pediatric PBPK model was then developed by incorporating age-specific parameters into the adult PBPK model, including parameters relevant for enalapril PK such as renal blood flow, renal and hepatic sizes, and values for relative CES 1 expression across the pediatric age [42,43]. Various simulations were performed with the pediatric PBPK model to predict enalapril and enalaprilat exposure in different age groups of virtual children and with different doses of enalapril: a total dose of 0.143 mg/kg, which is equivalent to an enalapril dose of 10 mg in an adult, and a dose of 1 × 0.25 mg ODMT in comparison to dosing regimens that are used currently on a mg/kg basis. This model was used to avoid excessive serum concentrations. A dose-banding regimen was developed, whereby whole numbers of 0.25 mg or 1.0 mg ODMTs could be administered to the patients, depending on their age and weight, which were predicted to lead to similar enalapril and enalaprilat exposures as those seen in adults for a start dose of 2.5 mg and a maintenance dose of 20 mg [44]. It was recognized that the model-based starting doses for neonates and infants using the 0.25 mg ODMTs might result in a slightly higher exposure than the adult start dose of 2.5 mg, although they were less than the dose at which renal failure has been reported in the literature case studies (0.1 mg/kg) [45,46,47,48]. Therefore, clinicians were given the opportunity to administer a lower starting dose, if required, by dispersing the 0.25 mg ODMT in a 10 mL oral syringe with water and administering a specified dispersion volume. For patient safety reasons and to allow the physician optimal dose individualization in this vulnerable population, the provided dosing regimen offered a lower initial dose in the form of a 10% or 50% dispersion of 0.25 mg ODMT up to an age of 6 months, although from the predicted exposure this lower dose was only necessary up to the age of 3 months. The PBPK model development with the adult pharmacokinetic data, physiological data, in vitro and preclinical data, and enalapril/enalaprilat specific information and physicochemical properties, such as molecular weight, lipophilicity, clearance, solubility, and intestinal permeability and some results of simulations performed using the PBPK model can be found in more detail in the supplement (File S1) and the dosing regimen in the supplementary materials (Table S3).

The dose ranges were not outside the ranges used in children older than 1 month of age, according to published data and routine use [6,7,21].

#### 2.3.4. Selection and Timing of Dose for Each Patient

Patients were initially administered with an initial dose (first morning dose) of enalapril under blood pressure monitoring for 8 h to identify a potentially extensive blood pressure decrease. If this dose was well tolerated, the patient either received this dose as the first dose for the next 1–6 days, or if the child was 3 years or older, dosing started in the evening with a second administration of this dose to achieve the full first dose. This first dose was up-titrated until the envisaged target dose and/or the maximum tolerated dose was reached. Patients with ACEI pretreatment were switched to a clinically comparable enalapril dose and from there potentially up-titrated to the maximum tolerated dose, according to the investigator’s clinical judgement and prescription.

Patients naïve to ACEI were up-titrated as follows according to the investigator’s judgement and prescription. For children aged 1 to younger than 6 months (2.5 and 7 kg) the recommended initial dose and first dose was 1 × 0.25 mg enalapril ODMT. Very young and low weight patients, for whom the investigator considered an initial dose of 1 × 0.25 mg enalapril ODMT to be too high, received a lower initial dose and first dose, such as 0.025 mg enalapril, 10% of 1 × 0.25 mg ODMT (0.01 mg/kg/day, diluted as 1 daily dose), or 0.125 mg enalapril (50% of 1 × 0.25 mg ODMT), up to a maximum of 4 × 0.25 mg ODMT (0.4 mg/kg/day in 2 daily doses). For children aged 6 months to younger than 3 years (8–15 kg), the recommended initial dose and first dose was 1 × 0.25 mg ODMT (once daily). For children aged 3 to less than 8 years (16–25 kg), the recommended initial dose was 1 × 0.25 mg ODMT, and the first dose was 2 × 0.25 mg ODMT (twice daily). For children aged 8 to less than 12 years (26–40 kg), the recommended initial dose was 0.5 mg ODMT, and the first dose was 2 × 0.5 mg ODMT once daily. Dose escalation was recommended by increasing the dose by 1 ODMT per dose level. The dose escalation interval was recommended to be 1 week. However, if the patient’s health condition required more rapid up-titration, the dose was increased on a daily basis at the investigator’s discretion if blood pressure, serum creatinine, and potassium results under the former dose permitted. The dosing was based on the response to the enalapril dose by obtaining the safety parameters of blood pressure, kidney function (creatinine), and potassium. Based on these parameters, a decision was taken to up-titrate, maintain, down-titrate, or stop enalapril. In case of a need for down-titration or stopping the dose, a reevaluation between 48 h was foreseen; otherwise, reevaluation was conducted after 1 week or less. In small children, the investigator could consider a routine assessment ≤ 48 h. Up-titration to the maximum tolerated dose was attempted for the first 5 weeks or less, according to the dosing scheme and dose titration scheme. Patients under ongoing ACEI treatment were switched to a clinically comparable enalapril dose of the dosing regimen and from there potentially up-titrated to the maximum tolerated dose according to the investigator’s clinical judgement and prescription. The dosing schedule is depicted in Table S1.

#### 2.3.5. Treatment Adherence

Parents were asked to fill-in a diary noting whether the morning and evening dose (if appropriate) had been administered to the child and at what time. To ensure that the patient had complied with the medication regimen, the returned medication was checked and counted by the designated site staff, and the returned amount recorded in a patient drug accountability log. If the records showed that the diary had not been correctly completed, or the patient was using less or more than the required amount of medication, the parent was informed about the child’s nonadherence, and detailed instructions were provided on how to ensure correct administration for the remainder of the study. The investigator entered the data on treatment adherence in the electronic case report form at each study visit, capturing the data from the previous week.

#### 2.3.6. Prior and Concomitant Therapy

Other DCM medications were permitted at the discretion of the investigator. These included but were not limited to diuretics, beta-blockers, digoxin, and mineralocorticoid receptor antagonists; acetylsalicylic acid for antiplatelet therapy only; or paracetamol. The following concomitant medication were not permitted: dual ACEI therapy, renin inhibitors, angiotensin II antagonists, and NSAIDs (including ibuprofen).

### 2.4. Pharmacokinetic Sampling and Pharmacokinetic Assessment

#### 2.4.1. Blood Sampling, Sample Preparation, Storage, and Transport of Samples

The primary outcome of the pharmacokinetic bridging studies was the exposure of enalapril and enalaprilat in children with heart failure determined as AUC within a dosing interval of 12 h, maximal plasma concentrations (Cmax), and time to maximal plasma concentration (tmax). In ACEI-treatment naïve patients, a pharmacokinetic profile was sampled either at the initial dose visit or at any visit under steady state conditions during the 8 weeks. In patients pretreated with an ACEI and in patients with an initial dose of 10% or 50% of ODMT 0.25 mg (dispersed dose, 0.025 mg or 0.125 mg, respectively), the pharmacokinetic profile could be sampled at any study visit performed after at least 7 days under the selected maintenance dose. For the pharmacokinetic profile sampling day, all children were allowed to have breakfast prior to taking the study medication. Serial blood samples were collected for 12 h after administration of enalapril. For blood sampling of enalapril and enalaprilat concentrations, serum collection tubes were applied. Where possible, at all sampling points, 1.2 mL S-monovettes (Sarstedt, Nuembrecht, Germany) were utilized. The selected devices allowed for either aspiration or vacuum collection of the blood. In case of very small vessels, in which blood collection by vacuum or aspiration technique appeared improper, the alternative blood collection devices were 1.3 mL serum micro tubes. This device enabled blood collection by blood dripping.

Site staff dedicated to the on-ward sample preparation procedure were intensively trained to ensure proper handling of the PK blood samples, according to the instructions provided in the sample collection manual [49,50]. The filled blood collection tubes were inverted multiple times and rested (allowed to stand) for at least 10 min in an upright position to allow for sufficient clotting, and then centrifuged and transferred into cryo tubes. The pipetted supernatant was snap-frozen before the serum samples were stored at 80 °C or colder until shipment of the frozen cryo tubes to the Heinrich-Heine Universität Düsseldorf, Institute of Clinical Pharmacy and Pharmacotherapy, Düsseldorf, Germany.

#### 2.4.2. Determination of Enalapril and Enalaprilat

For determination of the PK of enalapril and enalaprilat, liquid chromatography-triple quadrupole tandem mass spectrometry followed by solid-phase extraction of serum samples was applied (Shimadzu HPLC 10 [Shimadzu, Duisburg, Germany] coupled with AB Sciex API 2000 mass spectrometer [Sciex, Darmstadt, Germany]) [27,38]. The ion transitions were mass-to-charge ratio (*m*/*z*) 377.2 to 234.2 *m*/*z* for enalapril, 349.1 to 206.1 *m*/*z* for enalaprilat, and 425.3 *m*/*z* to 351.2 *m*/*z* for the internal standard benazepril. Moreover, the applied bioanalytical method was characterized by a small sample volume of 50 µL of serum encompassing a calibration range from 0.195–200 ng/mL for enalapril and 0.180–180 ng/mL for enalaprilat. The method was fully validated according to EMA and FDA bioanalytical method validation guidelines by using the European Pharmacopoeia Reference Standard for enalapril and enalaprilat. Obtained mean accuracy values for enalapril ranged from 92.1% to 108.4% of the nominal concentration at the lower limit of quantification (LLOQ), from 91.6% to 100.2% at the lowest concentration level, from 94.3% to 100.4% at the medium level, and from 92.6% to 98% at the upper limit of quantification (ULOQ). The time-different intermediate precision varied between 5.0% to 9.5% across all concentration levels and was subsequently well within the guideline limits of ±15% (±20% at LLOQ). Regarding the active metabolite enalaprilat, the mean accuracy values ranged as follows. At the LLOQ, the mean accuracy ranged from 88.0% to 105.5%, 90.2% to 98.8% at the low concentration, 94.0% to 100.9% at the medium level, and 93.2% to 106.4% at the ULOQ. Study samples measured below the LLOQ were set to 0, and samples above the ULOQ were remeasured after dilution [51].

#### 2.4.3. Data Analysis

The exposure of enalapril and its active metabolite enalaprilat in young children (1 month to under 12 years of age) were determined using descriptive PK investigations of the area under the concentration–time curve (AUC) from 0 h to the time of the last sampling point and maximal concentration (Cmax) and the time to maximal measured concentrations (tmax). This primary endpoint was depicted to obtain pediatric PK data of enalapril and its active metabolite enalaprilat in patients treated with enalapril ODMTs to describe the dose exposure in the pediatric population with DCM and CHD.

#### 2.4.4. Noncompartmental Analysis (NCA)

Enalapril and enalaprilat NCA were performed on the full serum profiles of patients taken at one visit. Separate NCA analyses were conducted for naïve patients and patients pretreated with ACE inhibitors (ACEIs), during which a full serum profile was performed at steady state. Steady state is defined as treatment with ODMTs after more than 7 days after switching patients from any ACEI pretreatment to the study treatment with enalapril ODMTs at a fixed dose level. The full serum profiles in naïve patients were performed on the first day of enalapril ODMT treatment. The full PK profiles of naïve patients who had received the first dose consisted of six serum samples taken at 0 h (predose) and 1 h, 2 h, 4 h, 6 h, and 12 h after dose administration. The full PK profile of patients taken at steady-state dosing consisted of five serum samples, taken at 0 h, 1 h, 2 h, 4 h, 6 h, and 12 h after dose administration.

PK parameters Cmax (maximum observed concentration), tmax (time of maximum observed concentration), and AUC0–12 (area under the concentration–time curve calculated using the trapezoidal rule within the dose interval of 12 h; AUC0–12 = AUCtau) were calculated using noncompartmental procedures. For statistical comparison of PK parameters among the limited number of patients and the patients groups the AUC within the dosing interval of the naïve patients were converted into the AUC at steady state (AUCtau,ss) according to Brett [52]. For this, an accumulation factor (R) was used as 1.5 for enalaprilat and 1 for enalapril. The factors were based on expected terminal half-life of 6–10 h for enalaprilat and less than 4 h for enalapril [5,6]. Based on the half-life the accumulation factor was calculated by R = 1/(1 − 2^−eps^), where eps is the ratio of dose interval and half-life and as R is equal to AUCss,tau/AUC0-tau, it can be used to convert the AUC after single dose of naïve patients into AUC at steady state of patients with only a pharmacokinetic profile at steady. It was evaluated that by using the different half-lives within the ranges, the converted AUCs at steady state did not change substantially. Since the pharmacokinetic profile had a restricted time window of only 12 h and an only limited set of data points, the calculation of the AUC within the dosing interval of naïve patients was more robust to use than the approximation of the terminal half-life.

Cmax was directly obtained from the concentration-vs.-time data as the maximum observed concentration in a profile, and tmax at steady state (tmax,ss) was the time at which Cmax (Cmax,ss) was observed. Similar to the AUC calculation, the Cmax within a dose interval was converted into Cmax at steady state using the accumulation factors of 1.5 for enalaprilat and 1 for enalapril.

### 2.5. Glomerular Filtration Rate

Glomerular filtration rate (GFR) in 4 centers was calculated using the Schwartz Equation (1), whereas F was dependent on the age of the patient (F = 0.45 for age up to 12 months, F = 0.55 for age older than 1 year) [53,54].

GFR = F∙Height/Serum creatinine(1)

In the 5th center GFR was calculated according to Equation (2)

GFR = 40.7(Height/Serum creatinine)^0.64^∙((30 × 0.357)/Blood Urea Nitrogen)^0.202^. (2)

Because of missing blood urea nitrogen and serum creatinine data for 1 patient, GFR was substituted with 100 mL/min for this patient.

### 2.6. Statistics

Calculations were performed using the software R, version 3.5.1. The analysis of variance (ANOVA) was carried out with software SAS. For the purpose of validation, calculations were also performed using the software WinNonlin^®^ (Pharsight Certara Princeton, NJ, USA; version 8.0). Furthermore, the parameters of noncompartmental PK were normalized by dose and body weight with abbreviations such as Cmax,ss,norm for Cmax,ss and AUCss,norm for AUCtau,ss. The following statistics were calculated for each of the sampling points as well as the calculated PK parameters: arithmetic mean, standard deviation, coefficient of variation, minimum value, median, maximum value, and number of measurements.

Descriptive statistics for plasma concentrations were presented over time by age group. Additionally, an analysis using plasma concentrations normalized by body weight as well as subgroup analyses by age group were provided. First step of the analysis was the analysis of variance with group parameter age group to elucidate, if the variance is different over the three age groups. For subgroup analysis, a *t*-test was used to compare means and their variances. Analysis for Cmax and AUC were carried out after natural log-transformation of the calculated parameters. Because of multiple testing significance level were adjusted by Bonferoni means *p* < 0.05, if two groups were compared but *p* < 0.0167, if 3 groups are compared. Values of zero were not included in the analysis. According to the analysis plan, concentrations below the lower limit of quantification were substituted by zero. The age groups tested were: (1) younger than 12 months of age; (2) between 1 and 6 years of age; and (3) between 6 and 12 years of age. All analyses were performed using validated R programs. The PK data analysis was conducted at the Institute of Clinical Pharmacy and Pharmacotherapy, Heinrich-Heine Universität Düsseldorf, Germany.

### 2.7. Safety Data

The safety, particularly the renal safety, of enalapril ODMTs in children and characterization of the dose–safety relationship from a starting dose to an optimal maintenance dose were secondary study outcomes. For safety monitoring at each visit, the patients’ parents were asked about AEs (adverse events) using nonleading questions. Additionally, a patient diary was used to record any clinical symptoms occurring between the visits. Furthermore, blood pressure; renal function; occurrence of cough; occurrence of angioedema; and a safety laboratory including hematology parameters, serum potassium, BUN, creatinine, and urinary micro albumin were assessed regularly. A Data and Safety Monitoring Board was established to supervise safety and other trial activities [8].

## 3. Results

### 3.1. Patient Characteristics

One hundred and two patients were enrolled in the study. Of these, 13 patients did not fulfill the eligibility criteria for the full pharmacokinetic profile, which was the study’s primary outcome. Therefore, only 89 patients were included in the per-protocol analysis. Of these, 26 patients had heart failure due to DCM, and 63 patients had heart failure due to CHD.

#### 3.1.1. Demographic Characteristics

Most of the patients (74%) were younger than 1 year of age, with the youngest patient being 23 days of age. As per the study’s inclusion criteria, newborns were included in only the patient group with CHD. Additionally, the DCM patient group included patients from 6 years to under 12 years of age. The different age distributions of the DCM and CHD patient groups were also reflected by differences in body weight, height, and BSA, as indicated in Table 1. Concerning gender, 55% of the total population was male, with a slightly higher percentage of male patients in the DCM cohort (65%) than in the CHD cohort (51%).

#### 3.1.2. Medical History of Patients with DCM and CHD

The cause of left ventricular systolic dysfunction in the cohort of patients with DCM was classified as idiopathic in the majority of cases (*n* = 21). Alström syndrome had been diagnosed in two cases, and another two cases were classified as familial/genetic. Other causes for left ventricular systolic dysfunction were related to coronary artery problems resulting from anomalous left coronary artery from the pulmonary artery after surgical repair (*n* = 3), myocardial infarction after arterial switch operation for simple transposition of the great arteries (*n* = 1), myocardial infarction in left coronary artery originating from the right coronary cusp after stent implantation after surgical correction of severe aortic valve stenosis (*n* = 1; Ross operation), and miscellaneous other reasons (*n* = 2). Among all patients with CHD, 46% were congenital, familial, or genetic disorders. Ten percent of patients were diagnosed as having CHD due to surgical and medical procedures, and 13% were diagnosed with respiratory, thoracic, and mediastinal disorders. The congenital defects of all patients resulted in high cardiac output. The most frequent diagnosis was ventricular septal defect. Of all patients, 50% had ventricular and atrial septal defects, 21% had isolated ventricular septal defect, and 11.4% had Down syndrome (trisomy 21). Although it was not mentioned in the exclusion criteria explicitly, the treatment of enalapril in breastfeeding mothers was either contraindicated or not recommended in the countries where the study was performed. Thus, interference of enalapril from breastfeeding mothers could be excluded.

#### 3.1.3. Enalapril Dosing Distribution

Table 1 also provides the mean dosing per body weight in the overall patient population, by cohorts, and by age groups at the time point when the full pharmacokinetic profiles were performed. Most of the DCM patients were pretreated with enalapril or captopril and switched to the enalapril minitablets. In contrast, the majority of CHD patients were naïve to ACEI treatment. For naïve patients, the full pharmacokinetic profile was performed on the first day of treatment. For switched patients, the full pharmacokinetic profile was performed at steady state after achieving the maintenance dose. Therefore, the mean doses presented in Table 1 are not comparable among the patient cohorts and age groups.

#### 3.1.4. Pretreatment and Concomitant Medication of Patients with DCM

The large majority of the children had been pretreated with an ACEI (*n* = 29, 91%); enalapril and captopril were the only ACEIs used. In children below the age of 6 years, enalapril and captopril use were equally distributed, whereas in children 6 years and older, enalapril was used exclusively. All these children were switched to equivalent doses of the enalapril ODMT. After switching to ODMTs, only 1 of 29 patients was up-titrated. Three children were naïve to ACEI and were up-titrated on enalapril ODMTs according to the dosing scheme. The ultimate dose reached in children ranged from 0.08 to 0.18 (mean and median 0.13) mg/kg/dose. Four patients discontinued the study prematurely, and all these patients were switched back to equivalent doses of commercial ACEIs. Their reason for trial discontinuation was withdrawal of consent by the family. In all cases, this withdrawal was related to the burden of study visits and/or venous blood withdrawals.

#### 3.1.5. Pretreatment and Concomitant Medication of Patients with CHD

More than half of the evaluable patients (*n* = 32) were ACEI naïve, and all of them were under 12 months old. The rest of the patients had been pretreated with an ACEI. Enalapril and captopril were the only ACEIs used as pretreatment. All ACEI-pretreated children were switched to a dose of enalapril ODMT, the majority to equivalent doses of enalapril. Children who were naïve to ACEI were up-titrated on enalapril ODMTs according to the protocol-provided dosing scheme or the physician’s clinical judgement.

#### 3.1.6. Renal Status

None of the patients had renal insufficiency. The glomerular filtration rates across age groups were comparable.

### 3.2. Pharmacokinetics of Enalapril and Enalaprilat

**Figure 1:**
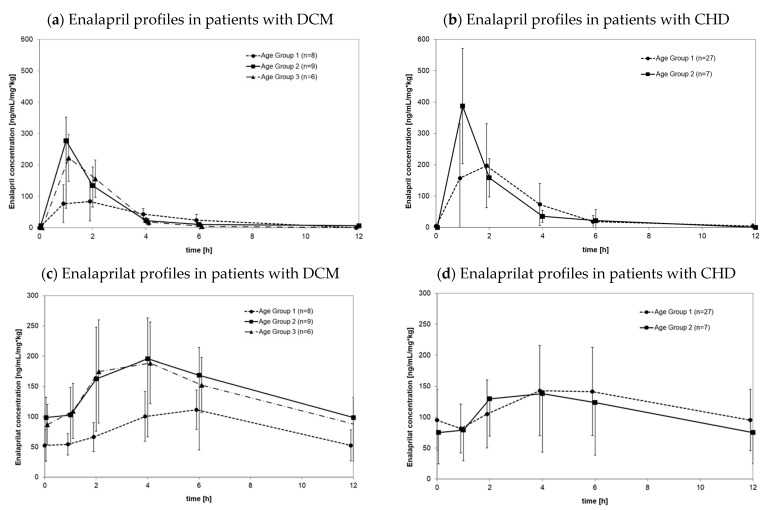
Enalapril (**a**,**b**) and enalaprilat (**c**,**d**) profiles of plasma concentrations over time with serum samples normalized by dose and bodyweight in steady state conditions in patients with heart failure due to dilated cardiomyopathy (**a**,**c**, *n* = 23) and congenital heart disease (**b**,**d**, *n* = 34) where profiles were measured under steady state conditions. Profiles were grouped according to age: Age group 1: 1 day to <1 year; Age group 2: 1 year to <6 years; age group 3: 6 years to <12 years.

Figure 1a depicts the mean plasma concentration profiles of enalapril concentrations in serum samples normalized by dose and body weight at steady state in the investigated pediatric population according to age groups independent of disease cohorts. Enalapril concentrations were highly variable at all investigated time points and mean concentrations did not differ substantially between age groups. Time of maximal concentrations, however, seemed to peak in enalapril about 1 h later in the youngest age group than in the older age groups. Figure 1b depicts the corresponding mean enalaprilat concentrations in serum samples normalized by dose and body weight at steady state in the investigated pediatric population by age group. Similar to enalapril, enalaprilat serum concentrations were highly variable and peaked later in the youngest age group than in older age groups.

Table 2 presents an overview of the calculated enalapril and enalaprilat pharmacokinetic parameters as NCA results concerning dose-normalized maximal plasma concentrations, exposure, and time to achieve maximal plasma concentrations for the overall patient sample and the subsets of patients with DCM or CHD. Because of concentrations below the lower limit of quantification for one patient (ENA concentrations) and three patients (ENAAT concentrations), data for Cmax as well as AUC show a minimum value of zero. This was the result of the analysis plan were BLQs were substituted to zero. All of these patients received a very low dose (less than 0.055 mg/kg). The ANOVA for age group within PK parameters for all patients of the present study resulted in no differences for natural log-transformed Cmax and AUC but for tmax. In contrast, the corresponding ANOVA for DMC patients resulted in no differences for tmax but for age dependent difference of AUC of enalaprilat and Cmax of enalapril. Details of the ANOVA are presented in Table S4 of the supplement. Three patients with DCM (WP08) and 29 patients with CHD (WP09) were naïve patients and therefore, PK parameters Cmax as well as AUC were extrapolated to steady state. Because the accumulation factor for enalapril was 1, for enalapril no difference in values of this parameters are expected. For further details, the results of statistics of PK parameters for subset of naïve patients are provided with Table S5 of the supplement.

#### 3.2.1. Pharmacokinetics in Patients with DCM

Age-dependent differences tend to be evident when comparing the youngest and oldest age groups. There is a trend of enalapril exposure increased by a factor of 1.3 from the oldest age group to the youngest age group. Compared to other age groups, the maximum enalapril concentration increased by a factor of 2.5 for patients aged under 1 year. For these patients, the time to reach the maximum concentration was 2 h, but it was 1 h for both other age groups. Similar results were found for enalaprilat for exposure, Cmax, and time to reach Cmax. Compared to patients under 1 year of age, AUCtau,ss,norm was about 1.7 times higher in patients 6 years of age or older. The corresponding factor for Cmax was about 2.5, and the time to reach maximum concentration was 6 h for children aged 1 year or younger but 4 h for both other age groups.

#### 3.2.2. Pharmacokinetics in Patients with CHD

Exposure to enalapril showed no difference between the youngest age group 1 (<1 year of age) and the next-oldest age group (1 year up to <6 years of age). Compared to patients older than 1 year of age, the maximum concentration of enalapril increased by a factor of 1.8 in younger patients. In these patients, the time to reach Cmax was 2 h, compared to 1 h in patients older than 1 year of age. The exposure and maximum concentration of enalaprilat in patients with CHD were identical, but time to reach Cmax was 6 h for younger patients, compared to 4 h in patients 1 year of age or older.

Only for patients with CHD, neonates from day 0 and 27 days were recruited. In Table 3 the individual age, body weight of the three neonates with congenital heart defects below 28 days of age were listed. All three neonates were male with an age of 17, 24, and 25 days and a corresponding body weight of 4.35, 3.28, and 3.50 kg. Moreover, enalapril as well as enalaprilat pharmacokinetic parameters were listed individually. The values did not differ substantially in comparison to the youngest age group above 27 days of age. In this cohort, there was only one patient recruited younger than 20 days of age. In contrast, data from Nakamura et al. [6] presented two patients with three plasma concentrations profiles below 20 days of age and reported substantially higher PK parameters than in the older patients investigated in that study.

#### 3.2.3. Pharmacokinetic Differences between DCM Patients and CHD Patients

The pharmacokinetic parameters in the DCM and CHD patients were statistically compared in the patients aged 1 month to under 6 years of age, where the two cohorts overlapped (Table 4). The statistical comparison demonstrated that DCM patients showed a 50% lower exposure to enalapril, compared to CHD patients. The metabolism of the active metabolite enalaprilat, however, was the same for both groups. Moreover, the time to achieve maximal concentrations of enalapril and enalaprilat were similar. Details regarding these results are reported in Table 4.

#### 3.2.4. Body-Weight-Normalized Clearance of Enalapril and Enalaprilat as Functions of Age

**Figure 2:**
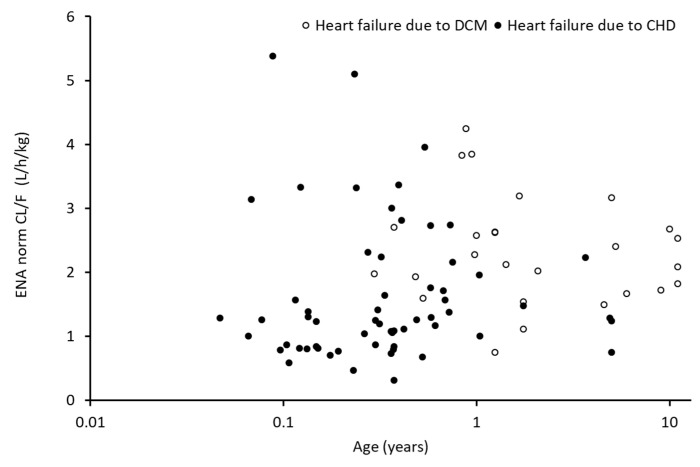
Body weight normalized apparent oral clearance of Enalapril (ENA) as function of age in patients with heart failure due to dilated cardiomyopathy (DCM, open circles, *n* = 25) and due to congenital heart disease (CHD, closed circle, *n* = 61).

The individual body-weight-normalized clearance of enalapril as functions of age is presented in Figure 2. Figure S7 presents an allome-tric scaling approach for enalaprilat as function of age. The filled circles indicate individual patients with heart failure due to CHD, and the open circles indicate individual patients with heart failure due to DCM. The individual clearances for enalapril and enalaprilat demonstrate high variability in both cohorts. The figure illustrates the different age ranges of the studied patients with CHD and DCM. Additionally, the figure illustrates that the high clearance variability dominates any potential influence of age.

### 3.3. Safety Evaluation

#### 3.3.1. Cohort of Patients with DCM

Out of the 32 patients included, 28 continued their participation and ODMT intake until the end-of-study visit. Four patients withdrew their consent because of the blood withdrawal burden and study visit frequency, thus terminating their participation in the clinical trial early. The administered enalapril ODMT doses were well tolerated. Eleven (34.5%) patients did not experience any AEs during their study participation. In total, 42 AEs occurred in 21 of the 32 (65.6%) patients enrolled in this study. Only 2 AEs were assessed as being possibly related to the intake of the enalapril minitablets. One event was vomiting after ODMT intake, and the second was a clinically relevant increase in microalbumin. One serious adverse event without a causal relationship with the ODMT occurred, namely syncope due to hypotension in the context of a gastrointestinal infection and hospitalization for observational purposes. No deaths occurred.

#### 3.3.2. Cohort of Patients with CHD

Out of the 70 patients, 56 patients continued their participation and ODMT intake until the end-of-study visit. Three patients withdrew their consent due to the blood withdrawal burden and study visit frequency, thus terminating their participation in the clinical trial early. In 11 patients, ODMT was stopped early for medical reasons that were unrelated to the ODMT: scheduled cardiac surgery in 7 patients, hyperkaliemia in 2, worsening of heart failure in 1, and hypotension in 1. The administered enalapril ODMT doses were tolerated well. Eighteen (25.7%) patients did not experience any AE during their study participation. In 52 (74.3%) of the 70 patients enrolled in this study, 118 AEs occurred. Only eight AEs (5.9%), were assessed as possibly, probably, or certainly related to ODMT intake. Eight (6.8%) AEs were of severe intensity, 32 (27.1%) AEs were of moderate intensity, and 78 AEs (66.1%) were of mild intensity. The most frequently observed AEs were cardiac operation (15 AEs, 12.7%), pyrexia (14 AEs, 11.9%), and rhinitis (9 AEs, 7.6%). All other AEs, described below, occurred less frequently. Five serious adverse events without causal relationship with the ODMT occurred; all but one (paresis of the right hemidiaphragm after cardiac surgery) resolved without sequelae. No deaths occurred. This section may be divided by subheadings. It should provide a concise and precise description of the experimental results, their interpretation, as well as the experimental conclusions that can be drawn.

## 4. Discussion

The primary objective of the LENA studies was to characterize the drug exposure and main pharmacokinetic parameters of enalapril and its metabolite enalaprilat in children with heart failure following the application of a novel formulation, enalapril ODMTs. Patients with two main etiologies of pediatric heart failure were included in the studies. One etiology group was the pediatric patient cohort with heart failure due to DCM, and the second group was the cohort with heart failure due to CHD. For both types of heart failure, the rate and extent of exposure of enalapril and its active metabolite enalaprilat were successfully characterized in the LENA studies. Furthermore, the rate and extent of enalapril and/or enalaprilat exposure were influenced by patients’ ages and types of heart failure. In these studies, when comparing the youngest age group with the oldest age group, the youngest patients had a delay in drug absorption and lower exposure than the oldest patient group. Furthermore, children with heart failure due to DCM had a 50% lower enalapril exposure than the children with CHD. Thus, age and disease interfere with the extent and rate of enalapril and enalaprilat exposure following the application of the novel formulation, the ODMTs.

The results presented here are the first pharmacokinetic data of enalapril and its metabolite enalaprilat after administration of a solid oral formulation to pediatric patients with heart disease and the first reported enalapril and enalaprilat pharmacokinetic data in children with heart failure due to DCM. For children with heart failure due to CHD; however, sparse pharmacokinetic data are available after application of an extemporaneously prepared suspension in 21 pediatric patients with heart failure [5,6] in an age range comparable to that of the LENA studies. Extemporaneous preparations, however, may cause imprecise dosing and additional variability in pharmacokinetic parameters. Moreover, not all pharmacokinetic parameters were reported. Enalapril and enalaprilat exposure were reported for only 11 patients [6]. For 10 patients, only maximum enalaprilat concentrations, but no exposure data [5]. As Nakamura et al. [6] and Lloyd et al. [5] investigated only children with heart failure due to CHD, the pharmacokinetics of pediatric patients with DCM had not been reported prior to the current study.

### 4.1. Pharmacokinetic Data in Children with Heart Failure Due to Congenital Heart Disease and Dilated Cardiomyopathy

The pharmacokinetic enalapril and enalaprilat exposure reported in 11 pediatric patients with heart failure due to CHD (older than 20 days to below 6.5 years of age) revealed similar rates and extents of exposure for enalapril and enalaprilat when comparing the dose- and body-weight-normalized reported Cmax and AUC values with the 63 patients’ LENA pharmacokinetic data [25l]. The PK data’s variability, however, was much smaller in the LENA data set than in the Nakamura data set. This may be due to the administration of the child-appropriate formulation of ODMTs in comparison to the more imprecise application of the extemporaneous suspension in the Nakamura study [55]. Pediatric patients with DCM revealed different enalapril exposure from that of pediatric patients with heart failure due to CHD. Enalapril exposure was about 50% lower in these patients. In contrast, enalaprilat exposure was similar in patients with both heart failure etiologies. Pathophysiologically, heart failure due to DCM results in lower cardiac output than most forms of heart failure due to CHD, which has been included in previous studies. Reduced cardiac output can induce systemic arterial hypoperfusion, neurohormonal activation, and venous congestion, which has reportedly altered drugs’ absorption, distribution, or renal and metabolic elimination [14,16,17,18]. Therefore, some of these factors might explain the lower enalapril exposure in DCM patients than in CHD patients. The active metabolite enalaprilat, however, did not show a lower exposure in the DCM cohort than in the CHD cohort. This finding indicates that the metabolism and elimination of enalaprilat were unaffected in DCM patients. In summary, we speculate that the pathophysiological difference in cardiac output between the patient cohorts affected enalapril exposure of but not that of the pharmacologically active metabolite enalaprilat. Thus, no clinical consequences should result from this issue. In the literature so far, differences in the pharmacokinetics of drugs between patients with heart failure due to DCM and those with heart failure due to CHD have not been reported. This may be explained by the fact that the numbers of heart failure studies investigating pharmacokinetic data and their numbers of pediatric patients typically are small. Moreover, the incidence and prevalence of heart failure is much smaller for children with DCM than for children with CHD. Thus, even if both etiologies of patients had been investigated, a statistical PK comparison between both patient groups would not have been possible because of the low patient number [22]. The largest study in children with heart disease due to DCM and CHD, published in 2007, presented trough concentrations of the beta receptor blocker carvedilol in 49 pediatric patients with heart failure [56]. Of those, 30 patients had heart failure due to DCM. Even in this investigation, trough concentrations were not differentiated based on heart failure etiologies, so information about etiology-based PK differences could not be gained.

### 4.2. Pharmacokinetics in Patients with Heart Failure and Age as Influencing Factors

Aside from disease etiology, patients’ ages influenced the extent and rate of enalapril and enalaprilat exposure. Across the LENA patient cohort, young patients demonstrated lower rates and lower extents of enalapril and enalaprilat exposure. These observations align with reports on a pediatric patient cohort with hypertension where enalapril and enalaprilat exposure were lower in infants than in older children and adolescents [7]. A phase I study in healthy adult subjects aged 22 to 47 years (median 24.4 years) using enalapril ODMTs as a reference formulation resulted in an enalapril exposure which was similar to the pediatric population. However, the exposure of the active metabolite enalaprilat was higher in the adult population compared to the pediatric population [24].

Age seems to influence enalapril absorption. In LENA, a prolonged time to reach maximal plasma concentrations of enalapril and enalaprilat was observed in the young age group, compared to those in the older age groups. The time to reach the maximum concentration of enalapril was 2 h for patients aged under 1 year but only 1 h for older age groups. Moreover, the time to reach the maximum concentration of enalaprilat was 6 h for patients aged under 1 year but only 4 h for the older age groups. Unfortunately, the patient cohort with hypertension in Wells et al. [7] were not able to demonstrate this because the blood sampling schedule in patients younger than 4 years of age had only a sparse sampling schedule of the predose sample and samples at 1, 4, and 8 h after dosing. In the LENA studies, a predose sample and 1-, 2-, 4-, and 6-h samples provided detailed information on the absorption phase of enalapril and enalaprilat; this information allowed for precise estimation of the time it took to reach maximum concentration in this young age group. The delayed enalapril absorption observed in the LENA study is also supported by the few data from Nakamura et al. [6], who presented three pediatric patients with CHD under 20 days of age and their times to reach maximal plasma enalapril and enalaprilat concentrations at 8 and 12 h [6]. Developmental changes in the gastrointestinal tract are well known and are reflected by changes in anatomical and physiological maturation. Gastric acid secretion, bile acid pool size, bile flow, and pancreatic enzymes such as lipases develop with age [9]. Furthermore, gastrointestinal transit times such as gastric emptying time, small intestinal transit time, and colonic transit time have been reported to be longer in neonates and infants, although feeding practices and diet also heavily influence gastrointestinal motility [9]. Since feeding practices and diets are profoundly different from those of older children, these factors may contribute to the differences in drug absorption [57,58]. In case of patients receiving the enalapril ODMTs, these gastrointestinal factors may have been responsible for the delayed absorption in neonates and infants. The ODMTs were placed in the patients’ cheek pouches, and a drink of the patients’/parents’ choice, such as breast milk, formula milk, cow milk, or water, was offered to facilitate swallowing and thus minimize the risk of choking. The patients did not need to follow a trial-specific diet or other food restrictions. The only requirement was that the parents were instructed not to mix the ODMTs into food. Therefore, there is no obvious single external or internal factor to explain the delayed time to reach maximum plasma concentrations observed in the young patient group.

### 4.3. Dosing Schedule of Enalapril Orodispersible Minitablets

Six serious AEs occurred in six patients. None were considered to be drug related, and none resulted in discontinuation of the study drug. Unexpected AEs not previously described in adults were not observed. Thus, the doses used in the present study appeared to be tolerated well and did not result in any significant drug-related AEs. The brief therapy duration in this 8-week study, however, would not permit identification of any delayed AEs. In the present study, children received solid formulations of ODMTs according to a dose-banding regimen based upon an age-appropriate physiologically based pharmacokinetic dosing schedule of enalapril that ranged from 0.25 to 16 mg per day. Calculating this dosing regimen for mean body weights of boys and girls in each age group resulted in enalapril doses of 0.06 to 0.27 mg/kg. Those doses are in the same range as the dosing regimens for the Nakamura study’s extemporaneous enalapril suspension, with 0.05 to 0.3 mg/kg [6] or the 0.07- to 0.14-mg/kg-dosed enalapril solution from the Wells study [7]. Based on these longstanding experiences in the use of enalapril in children with heart failure and those with hypertension of the same age range, it is unlikely that this dosing schedule might cause harm if applied in this patient cohort. However, accumulation of enalapril or enalaprilat might occur at higher doses if GFR is lower than 30 mL/min/1.73 m^2^. Numerous summaries of enalapril medicinal products’ characteristics report that renal insufficiency must be compensated for by lowering enalapril doses. All AEs and their relation to safety in the short and long terms as well as all collected secondary endpoints in relation to efficacy [8] will be reported in depth separately.

## Figures and Tables

**Table 1:** Demographics and body weight normalized dose in the pediatric population with heart failure due to dilated cardiomyopathy and congestive heart failure.

Patient Group	*n*	Gender (m)	Age (Years)	Body Weight (kg)	Dose (mg/kg)	Height (cm)	GFR * (mL/min)	BSA * (m^2^)
Total (ALL)	89	49 (55%)	0.422 (0.064–11.0)	5.93 (2.52–49.1)	0.0718 (0.0167–0.290)	65 (49.8–168.4)	118.8 (49.6–233.1)	0.327 (0.184–1.42)
1 day to <1 year	66	38 (58%)	0.363 (0.064–0.997)	4.93 (2.52–9.2)	0.06906 (0.0347–0.133)	61 (49.8–77.7)	115.8 (49.6–233.1)	0.285 (0.187–0.441)
1 year to <6 years	17	10 (59%)	1.75 (1.036–5.25)	11.9 (6.87–21)	0.09617 (0.0167–0.148)	85.6 (67.6–115.9)	125 (78.4–168.5	0.532 (0.370–0.795)
6 years to <12 years	6	4 (67%)	10.5 (6–11.02)	32.3 (17.8–49.1)	0.15658 (0.0396–0.290)	142.1 (111.9–168.4)	109.95 (94.2–151.1)	1.11 (0.74–1.41)
Dilated Cardiomyopathy	26	17 (65%)	1.54 0.296–11.02	9.83 5.48–49.1	0.107 0.027–0.290	81.5 62–168.4	112.7 71.8–151.1	0.463 0.311–1.41
1 months to <1 year	9	6 (67%)	0.844 (0.296–0.997)	8.9 (5.48–9.2)	0.107 (0.04–0.133)	73.5 (62–76.5)	100 (71.8–171.5)	0.429 (0.311–0.441)
1 year to <6 years	11	7 (64%)	1.75 (1.25–5.25)	10.3 (6.87–19.6)	0.1 (0.027–0.143)	84.4 (74.0–115.9)	114.5 (78.4–148.6)	0.491 (0.386–0.795)
6 years to <12 years	6	4 (67%)	10.5 (6–11.0)	32.3 (17.8–49.1)	0.157 (0.0396–0.290)	142.1 (111.9–168.4)	109.95 (94.2–151.1)	1.11 (0.74–1.41)
Congenital Heart Disease	63	32 (51%)	0.362 0.064–5	4.84 2.52–21.06	0.068 0.017–0.148	61.0 49.8–115	125.7 49.6–233.1	0.284 0.187–0.768
1 day to <1 year	56	29 (52%)	0.322 (0.064–0.751)	4.535 (2.52–8.78)	0.0651 (0.0347–0.133)	58.6 (49.8–77.7)	122.2 (49.6–233.1)	0.273 (0.187–0.435)
1 year to <6 years	7	3 (43%)	3.67 (1.04–5)	13.6 (7.3–21.0)	0.08 (0.017–0.148)	96.7 (67.5–115)	156.3 (108.2–168.5)	0.606 (0.370–0.768)

* GFR = Glomerular Filtration Rate; BSA = Body Surface Area.

**Table 2:** Pharmacokinetic parameters of enalapril and enalaprilat in pediatric patients with heart failure due to DCM and CHD.

Patient Group			Enalapril	Enalaprilat	Enalapril	Enalaprilat	Enalapril	Enalaprilat
		*n*	AUCtau, ss, norm (ng/mL·h/mg·kg)		Cmax, ss, norm (ng/mL/mg·kg)		tmax or tmax, ss (h)	
Total (All)		89	637.5	1166.4	231.6	138.5	1.98	5.95
			(0–3144.8)	(0–5451.9)	(0–760.8)	(0–719.5)	(0–5.95)	(0–12.2)
1 day to <1 year (Ia)		65	721.9	1111.7	219.4	128.8	2	6
			(0–3144.8)	(0–5451.9)	(0–603.5)	(0–719.5)	(0–5.95)	(0–12.2)
1 year to <6 years (Ib)		18	580.2	1272.2	279.2	139.7	1.03	4
			(313.0–1338.2)	(0–4468.2)	(78.9–279.2)	(0–516.3)	(1–4.08)	(0–12.02)
6 years to <12 years (Ic)		6	514.6	1618.7	240.95	187.8	1	3.92
			(373.1–597.8)	(872.6–1968.6)	(155.5–273.2)	(103.3–268)	(1–2)	(2–4)
p, Ia vs. Ib			0.849	0.4039	0.1038	0.3613	0.011 #	0.003 #
p, Ia vs. Ic			0.0809	0.0333	0.1851	0.1721	0.022	<0.0001 #
p, Ib vs. Ic			0.1476	0.3257	0.4547	0.5413	0.8683	0.5149
Dilated Cardiomyopathy		26	455.4	1194.4	148.0	129.2	1.51	4.56
			(235.5–1338.2)	(0–4468.2)	(44–760.8)	(0–516.3)	(0.93–4.17)	(0–12.0)
1 months to <1 year (IIa)		9	387.7	929.5	97.1	109.6	2.03	6
			(235.5–626.1)	(390.7–1569)	(44–219.4)	(66.8–187.7)	(0.93–4.17)	(4.03–6.08)
1 year to <6 years (IIb)		11	470.7	1285.4	225.3	148.8	1.03	4
			(313–1338.2)	(0–4468.2)	(78.9–760.8)	(0–516.3)	(1–4.08)	(0–12.0)
6 years to <12 years (IIc)		6	514.6	1618.7	240.95	187.8	1	3.92
			(373.1–597.8)	(872.6–1968.6)	(155.5–273.2)	(103.3–268)	(1–2)	(2–4)
p, IIa vs. IIb			0.1005	0.0212	0.0039 #	0.0213	0.0646	0.128
p, IIa vs. IIc			0.1018	0.0062 #	0.0005 #	0.015 #	0.0229	0.0003 #
p, IIb vs. IIc			0.7078	0.7708	0.8555	0.8426	0.6496	0.6443
Congenital Heart Disease		63	778.2	1166.1	260.3	142.2	1.98	6.0
			(0–3144.8)	(0–5451.9)	(0–603.5)	(0–719.5)	(0–5.95)	(0–12.2)
1 day to <1 year (IIIa)		56	783.7	1156.6	257.1	142.7	2	6
			(0–3144.8)	(0–5451.9)	(0–603.5)	(0–719.5)	(0–5.95)	(0–12.2)
1 year to <6 years (IIIb)		7	778.2	1258.9	471.9	130.5	1.03	4
			(448.8–1334.8)	(166.5–2706.9)	(105.1–564)	(81.8–289.3)	(1–2.1)	(2.07–6)
p, IIIa vs. IIIb			0.5217	0.8282	0.1021	0.9591	0.0026 #	0.0006 #

Data reported as median (range), statistical testing was performed by t-test were used to compare means and their variance, *p*-values are reported; DCM = Dilated Cardiomyopathy; CHD = Congenital Heart Disease, AUC = Area under the curve; Cmax = maximal serum concentrations; tmax = time of maximal serum concentrations; ss = steady state, norm = normalized. # significant based on p adjusted according to Bonferoni.

**Table 3:** Individual age, body weight and pharmacokinetic parameters of the four male neonates with congenital heart defects below 28 days of age.

Age	Body Weight	Enalapril	Enalaprilat	Enalapril	Enalaprilat	Enalapril	Enalaprilat
(days)	(kg)	AUCtau, ss, norm (ng/mL·h/mg·kg)		Cmax, ss, norm (ng/mL/mg·kg)		tmax or tmax, ss (h)	
17	4.35	775.3	259.5	1344.6	174.9	2.05	5.983
24	3.28	996	274.8	504.9	101.8	3.983	11.9
25	3.50	318.6	103.8	1111.7	152.5	1	5.983

**Table 4:** Comparison of enalapril and enalaprilat pharmacokinetic parameters in DCM and CHD patients of same age range.

		Enalapril	Enalaprilat	Enalapril	Enalaprilat	Enalapril	Enalaprilat
	*n*	AUCtau, ss, norm (ng/mL·h/mg·kg)		Cmax, ss, norm (ng/mL/mg·kg)		t-max or tmax, ss (h)	
Dilated Cardiomyopathy 1 months to <6 year	20	428.3	1040.1	136.4	120.4	1.99	5.37
		(235.5–1338.2)	(0–4468.2)	(44–760.8)	(0–516.3)	(0.93–4.17)	(0–12.02)
Congenital Heart Disease 1 months to <6 years	60	785.1	1166.3	261.0	142.1	1.98	6.0
		(0–3144.8)	(0–5451.9)	(0–603.5)	(0–719.5)	(0–5.95)	(0–12.2)
p DCM versus CHD		0.0025 #	0.4517	0.051	0.9543	0.7632	0.0095 #

Data reported as median (range), statistical testing was performed by t-test were used to compare means and their variance, *p*-values are reported DCM = Dilated Cardiomyopathy; CHD = Congenital Heart Disease, AUC = Area under the curve; Cmax = maximal serum concentrations; tmax = time of maximal serum concentrations; ss = steady state, norm = normalized, # significant based on p adjusted according to Bonferoni.

## Data Availability

Data supporting reported results were submitted to the EU commission Available online: https://cordis.europa.eu/project/id/602295/reporting/de (accessed on 26 May 2022).

## Supplementary Materials

The following supporting information can be downloaded at: https://www.mdpi.com/article/10.3390/pharmaceutics14061163/s1, File S1: Development of the physiologically based pharmacokinetic model and the dosing schedule. Table S1: Model input parameters of the coupled physiologically based pharmacokinetic enalapril/enalaprilat model. Figure S1. Com-parison of predicted and mean observed concentrations of oral enalapril and enalaprilat after a 20 mg dose of enalapril. Table S2: Comparison of the predicted and observed exposure (AUC0–t) and maximal concentration (C_max_) of the mean concentration time-curves of the adult PBPK enalapril and enalaprilat model. Figure S2. Ontogeny profile of CES 1. Figure S3. Simulations with the pe-diatric PBPK model to predict enalapril and enalaprilat exposure in different age groups of virtual children. Figure S4: Comparison of the predicted enalaprilat exposure (AUC0–48) in 4 different age groups. Figure S5: Weight normalized enalaprilat exposure (AUC0–24) across paediatric age after the proposed dose banding schedule (starting dose). Figure S6: Relative exposure simulations for the application of the dispersed ODMTs (10% of 0.25 mg = 0.025 mg enalapril/day or 50% of 0.25 mg enalapril= 0.125 mg enalapril/day).Table S3: Dosing schedule for enalapril orodispersible minitablets. Table S4: Results of ANOVA test for Age Group. Table S5: PK parameters for naïve patients. Figure S7: Allometric scaling of apparent oral clearance (normalized by body weight).
