# Supplementary material for: Enalapril and Enalaprilat Pharmacokinetics in Children with Heart Failure Due to Dilated Cardiomyopathy and Congestive Heart Failure after Administration of an Orodispersible Enalapril Minitablet (LENA-Studies)
Source: Pharmaceutics. 2022 May 30;14(6):1163. doi: 10.3390/pharmaceutics14061163 (PMC9228797; doi:10.3390/pharmaceutics14061163)
Supplement: Supplementary file 1 [file pharmaceutics-14-01163-s001.zip › pharmaceutics-1684600-Supplementary.pdf]

Supplement File S1 PBPK Model

Physiologically based pharmacokinetic modelling (PBPK) to determine the study´s dosing regimen

The study's dosing regimen was determined using modelling and simulation, that is, physiologically based pharmacokinetic simulation (PBPK, SIMCYP® v13.2) with the aim of achieving a similar exposure in the various pediatric age subsets to that achieved in adults. A similar approach as that described by Khalil [1S, 2S] was used to develop the enalapril/enalaprilat pediatric PBPK model. Data from the literature, including adult pharmacokinetic data, physiological data, in vitro and preclinical data, and enalapril/enalaprilat specific information and physicochemical properties, such as molecular weight, lipophilicity, clearance, solubility, and intestinal permeability, were collated (**Table S1**). The commercially available modelling software package (SIMCYP® v13.2) was used to provide the general model structure with a standard database of anatomical and physiological parameters for adults and children. Enalapril and enalaprilat specific data were incorporated to build first a coupled adult PBPK model for the prodrug and its metabolite before scaling it to children. A full PBPK model was used with enalapril, and a minimal PBPK model was used with enalaprilat. The Advanced Distribution and Absorption Model (ADAM) was used for the absorption of enalapril.

Table S1: Model input parameters of the coupled physiologically based pharmacokinetic enalapril/enalaprilat model

**Parameter**	**Enalapril**		**Enalaprilat**	
	**Model input**	**Reference**	**Model input**	**Reference**
Molecular weight (g/mol)	376.4462	Pubmed. CID 5388962	348.39	Pubmed. CID 5462501
LogP	0.67	Takagi 2006 [3S]	-0.74	Remko 2007 [4S]
pK_a values	3; 5.4	Kasim 2003 [5S]	3.13; 7.83	Drugbank.ca
Blood to plasma ratio	0.58	ADMET predictor[b]	0.56	ADMET predictor[b]
f_u	0.55	Sirianni and Pang 1998 [6S]	0.44	ADMET predictor[b]
Renal clearance (L/h)	25	Optimization*	7 [c]	Hockings 1986 [7S]
CES1 abundance (pmol/mg protein)	402	Sato 2012 [8S]	NVA	NVA
CES1 CL_{int} (µL/min/mg protein)	15.26	Optimization*	NVA	NVA
Additional systemic clearance (L/h)	8	Optimization*	NVA	NVA
Intestinal permeability (10^{-4} cm/s)	Regional [a]	Optimization*	NVA	NVA
Intrinsic solubility (mg/ml)	21	Avdeef 2001 [9S]	NVA	NVA

LogP= octanol-water partition coefficient; pK_a= acid dissociation constant; f_u= fraction unbound; CES1= Carboxylesterase; NVA= no value was assigned.

[a] Intestinal permeability was assigned as $3.5x10^{-4}$ cm/s in the jejunum and $1.4x10^{-4}$ cm/s in all other intestinal segments in order to account for the active transport by the peptide transporters. These values gave a fraction absorbed of >90%.

[b] Calculated values using the ADMET Predictor™ (V6.5, Simulations Plus, Inc., Lancaster, CA).

[c] Reference value is 6.10 L/h; however, values up to 9 L/h are reported in the literature.

* Obtained by optimization/fitting to the observed adult data

Full PBPK model was used with enalapril as a prediction method for tissue partition coefficients [10S, 11S, 12S]

Minimal PBPK model was used with enalaprilat (input V_{ss}=2.4 L/kg)

The Advanced Distribution and Absorption Model (ADAM) was used for the absorption of enalapril.

In order to evaluate the developed adult PBPK model, predicted data derived from the model were compared with results obtained from a single dose PK study in healthy adults [13S]. The results of this comparison are provided in **Figure S1**, from which it can be seen that the model was shown to be able to accurately reflect the behaviour of both enalapril and enalaprilat in the adult population.

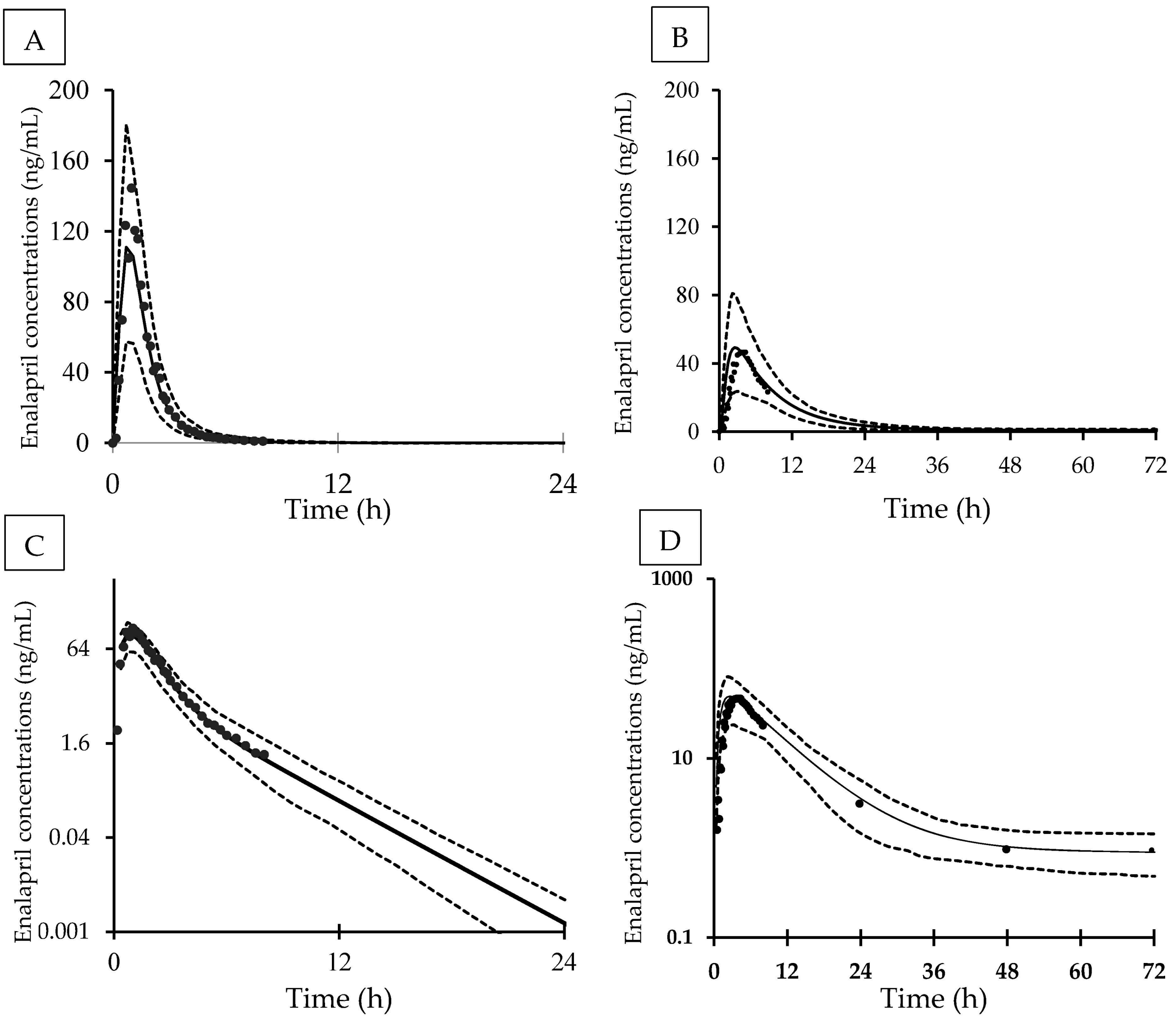

Figure S1. Comparison of predicted (lines; mean, 5th and 95th percentiles) and mean observed (dots) concentrations of oral enalapril (A, C) and enalaprilat (B, D) after a 20 mg dose of enalapril. Concentrations are shown on a linear scale (upper panel) and on a semi-logarithmic scale (lower panel). Simulations were performed using a PBPK model developed by SIMCYP® software. Observed data are obtained from a single dose PK study in healthy adult volunteers of 19-30 years old [13S].

The **Table S2** below show a comparison of the predicted and observed AUC0-t and Cmax. of the mean concentration time-curves.

Table S2: Comparison of the predicted and observed exposure (AUC0-t) and maximal concentration (Cmax) of the mean concentration time-curves of the adult PBPK enalapril and enalaprilat model

	Enalapril			Enalaprilat		
Parameter	Predicted	Observed	Relative percentage Error	Predicted	Observed	Relative percentage Error
AUC_{0-t} (ng/mL/h)	212.65	219.08	-2.9%	527.74	507.27	4.1%
C_{max} (ng/mL)	111.03	144.52	-23.2%	49.20	46.49	5.8%

The pediatric PBPK model was then developed by incorporating age-specific parameters into the adult PBPK model, including parameters relevant for enalapril PK such as renal blood flow, renal and hepatic sizes, and values for relative CES 1 expression across the pediatric age [14S, 15S]. The latter has been found to increase with age, as shown in **Figure S2.** Literature paediatric PK data were not incorporated as their reliability is questionable due to the lack of specificity of the analytical methodology used.

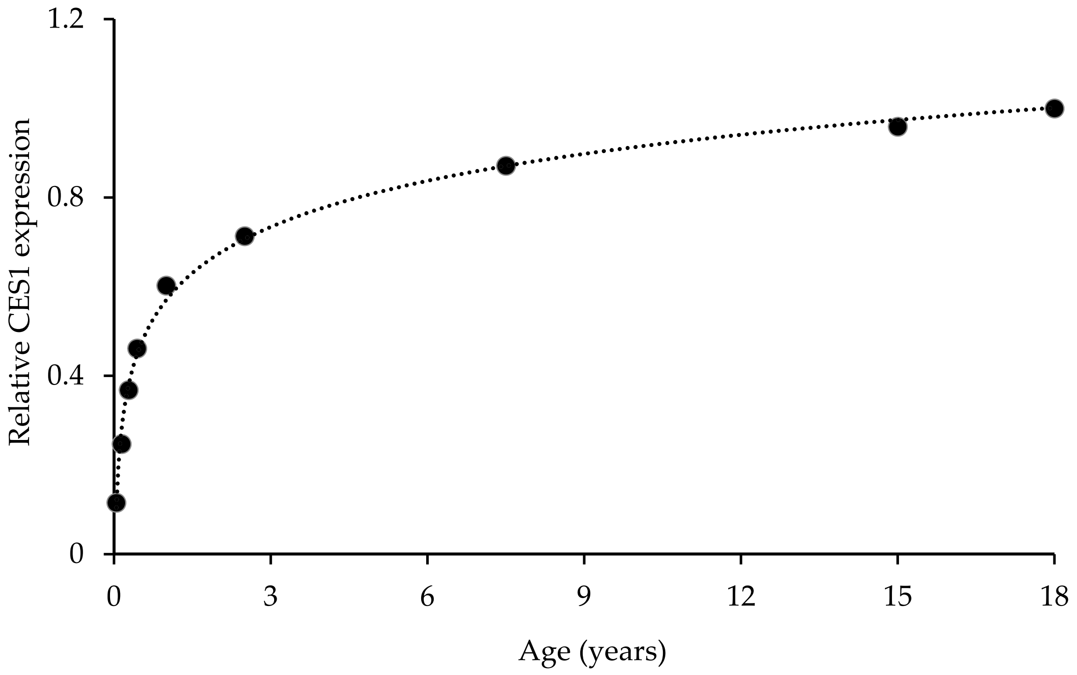

Figure S2. Ontogeny profile of CES 1. The incorporated ontogeny profile of CES 1 according to the collected information of relative CES1 expression in different paediatric ages [14S, 15S] using the following equation: $y = 0.1491\ln(x) + 0.5704$; $r^2 = 0.9964$.

Various simulations were performed with the pediatric PBPK model to predict enalapril and enalaprilat exposure in different age groups of virtual children and with different doses of enalapril: a total dose of 0.143 mg/kg, which is equivalent to an enalapril dose of 10 mg in an adult, and a dose of 1 x 0.25 mg ODMT in comparison to dosing regimens that are used currently on a mg/kg basis. This model was used to avoid excessive serum concentrations. Each age group consisted of 100 virtual children of both genders (50:50) of the respective age. The given dose was extrapolated from an adult dose of 10 mg enalapril in 70 kg subjects. The age of the adult reference population is 18-30 years, which is the age of the adult population [13S]. The results are presented in **Figure S3.**

A)

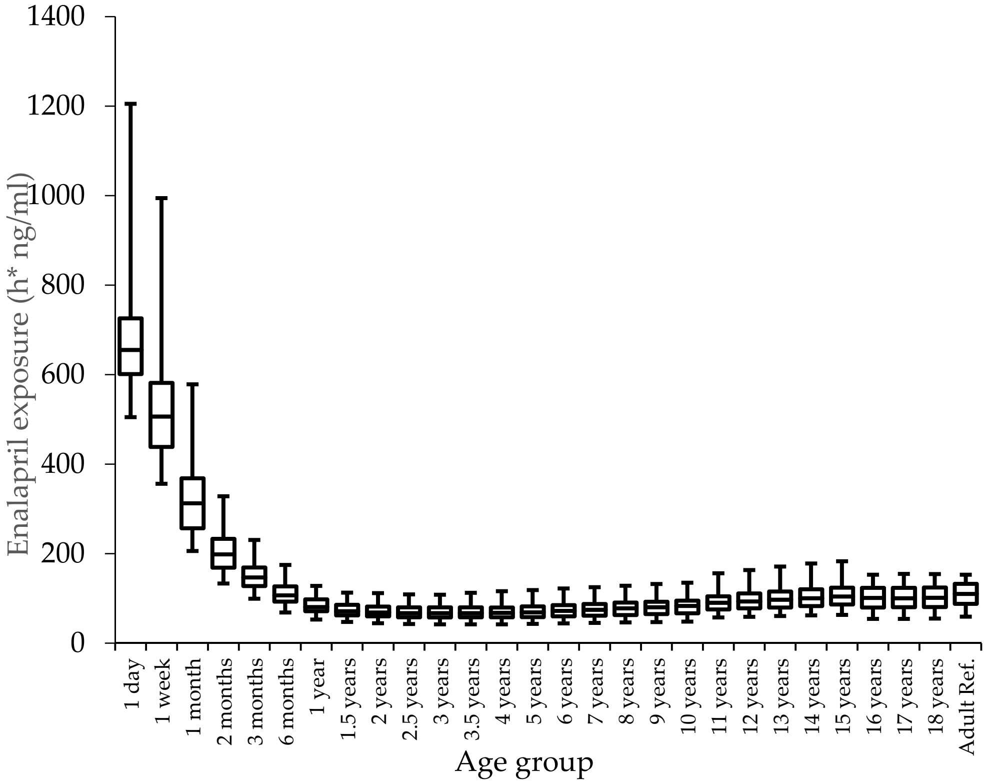

B)

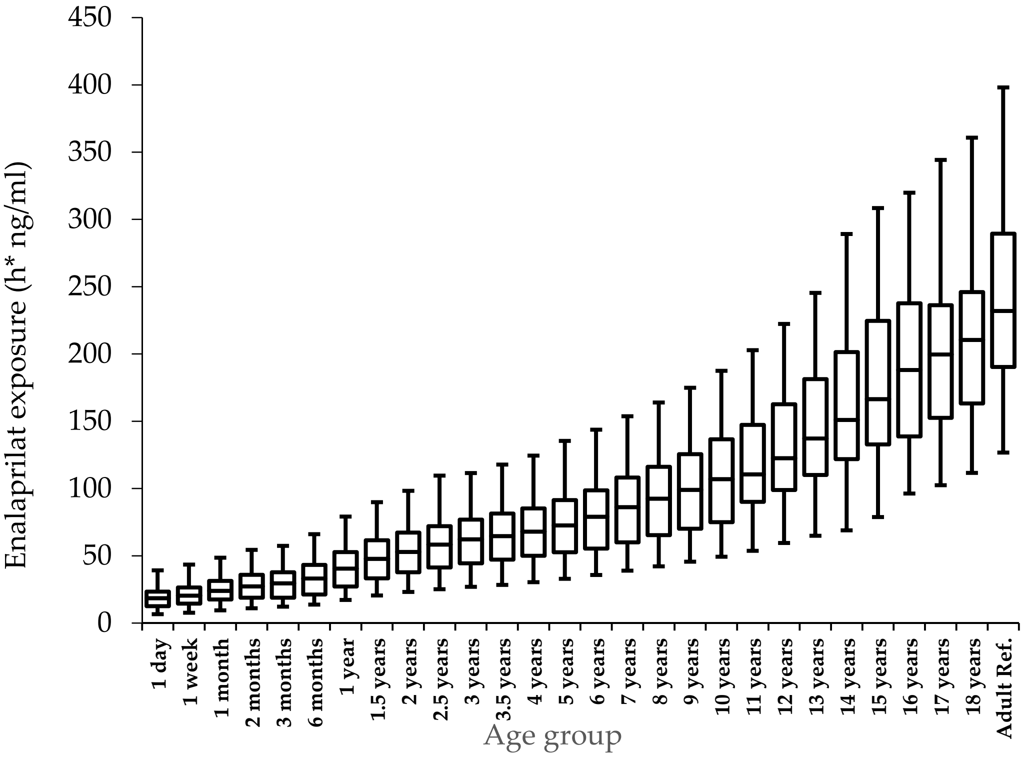

.

Figure S3: Enalapril (A) and enalaprilat (B) exposure ($AUC_{0\text{-}48}$) across the paediatric age after a 0.143 mg/kg enalapril dose. Comparison of the predicted enalapril exposure (AUC0-48) in different paediatric age groups after the administration of a unique total dose of 0.143 mg/kg using the developed PBPK model. The results are presented as boxplots including the 5th, 25th, 50th (median), 75th, and the 95th percentiles of the predicted drug exposure.

Paediatric PBPK model simulations

The paediatric PBPK model was used to perform a number of "what if" simulations in different age groups of virtual children in order to predict enalapril and enalaprilat exposure, with different doses of enalapril. Although doses of enalapril in adult heart failure patients are well defined, there is no consistency in the current literature regarding enalapril doses in paediatric patients, mainly due to its off label use. There is a concern that ACEIs can cause renal impairment, especially when taken at high doses. However, very low doses of enalapril may be ineffective. Out of the literature, where safety concerns had been discussed in clinical practice [16S, 17S], a low starting dose of enalapril (0.025 mg/kg) was used to compare the simulated exposure of enalaprilat after application of one dose of 0.25 mg orodispersible minitablet in to paediatric individuals from neonates up to adolescents of 18 years (**Figure S4**).

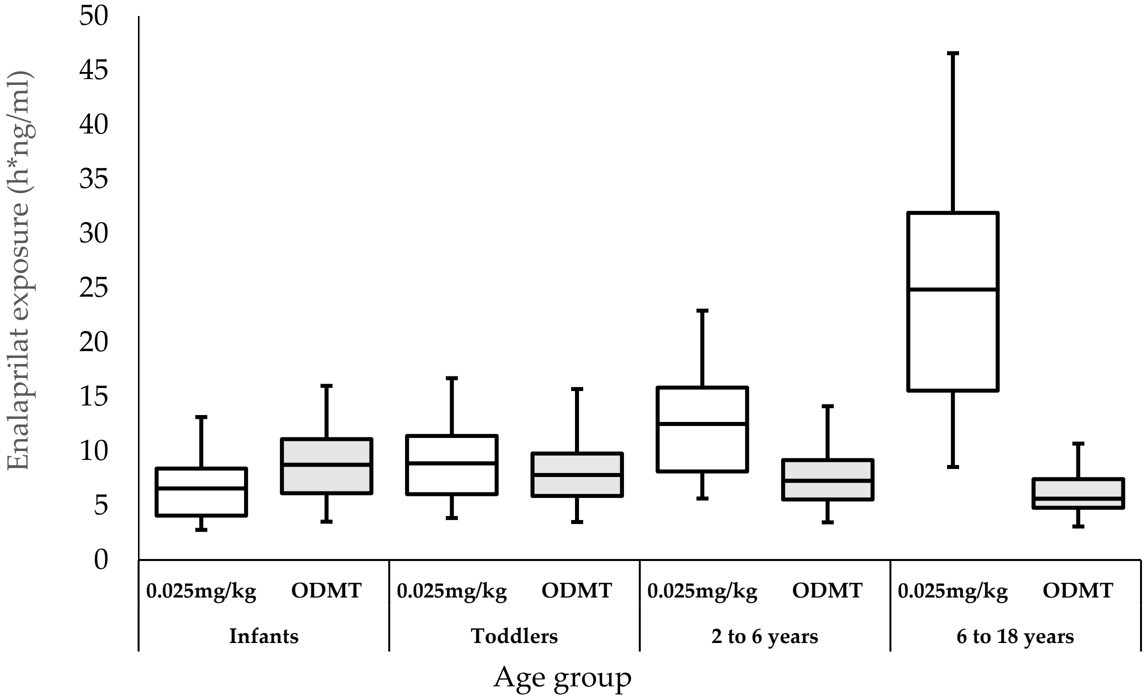

Figure S4: Comparison of the predicted enalaprilat exposure (AUC_{0-48}) in 4 different age groups (infants=28days to below 1 year; toddlers=1 year to below 2 years) after the administration of a proposed starting dose of 0.025 mg/kg enalapril dose or after the administration of a unique dose of 1 x 0.25 mg ODMT using the developed PBPK model.

Proposed dosing regimen for paediatric studies

The above simulations show distinct age dependent differences on drug exposure with high variability in young children. The data generated were used to derive a dosing regimen for the paediatric clinical studies. For that, a dose-banding regimen was developed (see **Table S3**), whereby whole numbers of 0.25 mg or 1.0 mg ODMTs could be administered to the patients, depending on their age and weight, which were predicted to lead to similar enalapril and enalaprilat exposures as those seen in adults for a start dose of 2.5 mg and a maintenance dose of 20 mg

Then, the PBPK model was used to simulate the potential exposure of enalaprilat in age groups up to 18 years of age. For comparison, also the predicted exposure in young adults (70 kg body weight) was simulated using a typical adult starting dose of 2.5 mg enalapril. Each age group consisted of 100

virtual children of both genders (50:50) of the respective age. Results are presented as boxplots including the 5th, 25th, 50th (median), 75th, and the 95th percentiles of the predicted drug exposure (**Figure S5**).

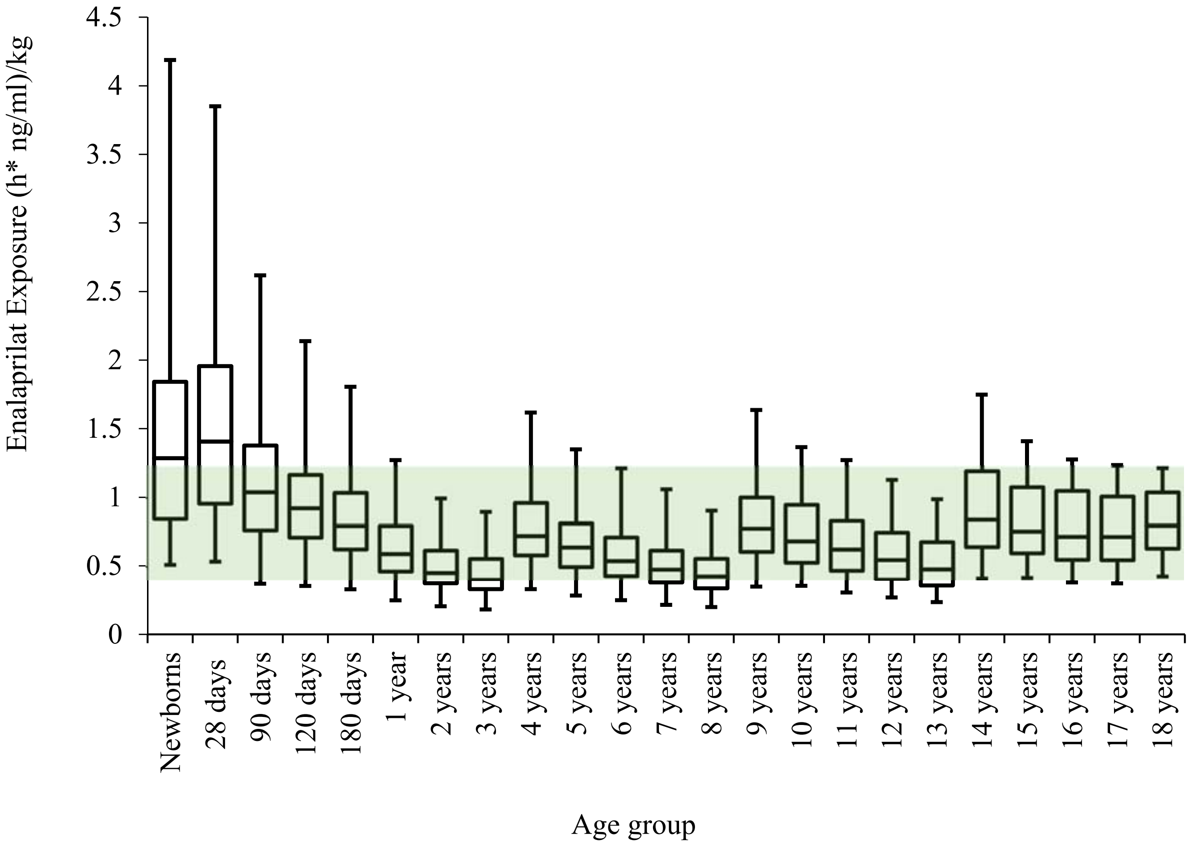

Figure S5: Weight normalized enalaprilat exposure (AUC0-24) across paediatric age after the proposed dose banding schedule (starting dose). Comparison of the predicted weight normalized enalaprilat exposure (AUC_{0-24}) by the PBPK model in different paediatric age groups after the administration of ODMT starting doses as described in the proposed dosing regimen. The grey shaded area indicates the range between the 5th and 95th percentiles of the predicted exposure in 18 year old young adults (reference group) that received the typical adult starting dose of one 2.5 mg enalapril tablet.

It could be seen that all age groups except the young ones fit very well into this adult exposure range. However, there was a high degree of variability in the predicted enalaprilat exposures in neonates and infants. It was recognized that the model-based starting doses for neonates and infants using the 0.25 mg ODMTs might result in a slightly higher exposure than the adult start dose of 2.5 mg. Therefore, clinicians were provided with the opportunity to administer a lower initial dose, if required (0.01 mg/kg/day enalapril). For patient safety reasons and to allow the physician optimal dose individualization in this vulnerable population, the provided dosing regimen offered a lower initial dose in the form of a 10% or 50% dispersion of 0.25 mg ODMT up to an age of 6 months, although from the predicted exposure this lower dose was only necessary up to the age of 3 months. **Figure S6** presents the exposure simulations for the application of the dispersed ODMTs (10% of 0.25 mg = 0.025 mg enalapril/day or 50% of 0.25 mg enalapril= 0.125 mg enalapril/day) in the proposed dose banding scheme for respected age or age group (AUC0-24).

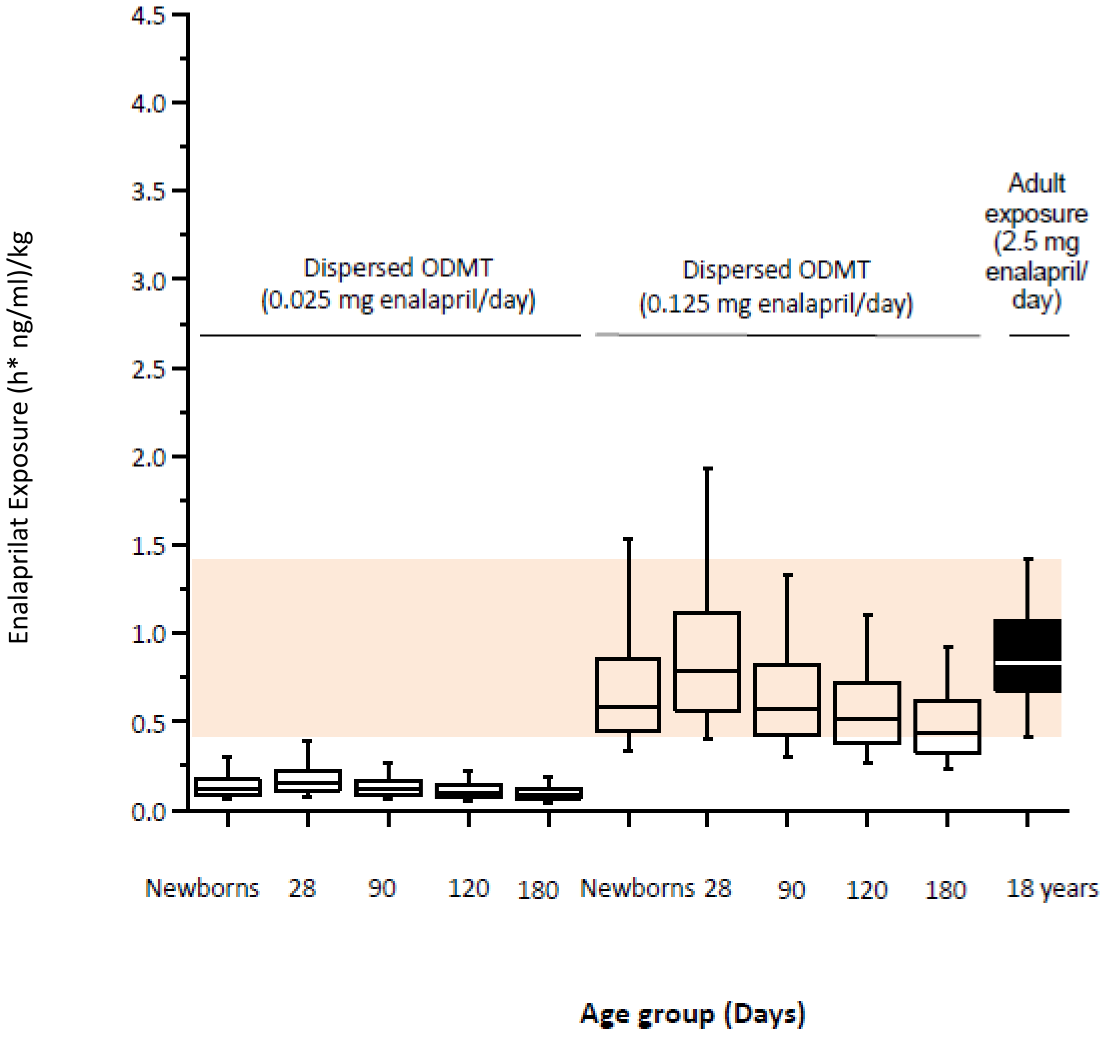

Figure S6: Relative exposure simulations for the application of the dispersed ODMTs (10% of 0.25 mg = 0.025 mg enalapril/day or 50% of 0.25 mg enalapril= 0.125 mg enalapril/day) in the proposed dose banding scheme for respected age or age group (AUC_{0-24}).

The development of physiology based pharmacokinetic modeling of the enalapril paediatric dosing scheme has been presented at the 50th Annual Meeting of the Association for European Paediatric and Congenital Cardiology (AEPC) in Rome June 1-4, 2016 (18S).

References

1S Khalil F, Laer S. Physiologically based pharmacokinetic modeling: Methodology, applications, and limitations with a focus on its role in pediatric drug development. J Biomed. Biotechnol. 2011; Article ID 907461, doi:10.1155/2011/907461.

2S Khalil F, Laer S. Physiologically based pharmacokinetic models in the prediction of oral drug exposure over the entire pediatric age range—Sotalol as a model drug. The AAPS Journal 2014;16:226-39

3S Takagi T, Ramachandran C, Bermejo M, Yamashita S, Yu LX, Amidon GL. A provisional biopharmaceutical classification of the top 200 oral drug products in the United States, Great Britain, Spain, and Japan. Molecular pharmaceutics. 2006 3:631-43

4S Remko M. Acidity, lipophilicity, solubility, absorption, and polar surface area of some ACE inhibitors. Chemical Papers. 2007; 61: 133-41

5S Kasim NA, Whitehouse M, Ramachandran C, Bermejo M, Lennernäs H, Hussain AS, Junginger HE, Stavchansky SA, Midha KK, Shah VP, Amidon GL. Molecular properties of WHO essential drugs and provisional biopharmaceutical classification. Molecular pharmaceutics. 2004; 1:85-96

6S Sirianni GL, Pang KS. Intracellular and not intraluminal esterolysis of enalapril in kidney. Studies with the single pass perfused nonfiltering rat kidney. Drug metabolism and disposition. 1998, 26:324-31

7S Hockings N, Ajayi AA, Reid IL. Age and the pharmacokinetics of angiotensin converting enzyme inhibitors enalapril and enalaprilat. British journal of clinical pharmacology. 1986 21:341-8

8S Sato Y, Miyashita A, Iwatsubo T, Usui T. Simultaneous absolute protein quantification of carboxylesterases 1 and 2 in human liver tissue fractions using liquid chromatography-tandem mass spectrometry. Drug metabolism and disposition. 2012, 40:1389-96

9S Avdeef A, Berger CM. pH-metric solubility. 3. Dissolution titration template method for solubility determination. European journal of pharmaceutical sciences. 2001, 14:281-91.

10S Rodgers T, Leahy D, Rowland M. Physiologically based pharmacokinetic modeling 1: predicting the tissue distribution of moderate-to-strong bases. J Pharm Sci 2005, 94: 1259–1276.

11S Rodgers T, Rowland M. Physiologically based pharmacokinetic modelling 2: predicting the tissue distribution of acids, very weak bases, neutrals and zwitterions. J Pharm Sci 2006, 95: 1238–1257.

12S Rodgers T, Rowland M. Mechanistic approaches to volume of distribution predictions: understanding the processes. Pharm Res 2007, 24: 918–933.

13S Burckhardt BB, Tins J, Ramusovic S, Läer S. Tailored Assays for Pharmacokinetic and Pharmacodynamic Investigations of Aliskiren and Enalapril in Children: An Application in Serum, Urine, and Saliva. J Pediatr Pharmacol Ther. 2015; 20:431-52.

14S Zhu HJ, Appel DI, Jiang Y, Markowitz JS. Age- and sex-related expression and activity of carboxylesterase 1 and 2 in mouse and human liver. Drug Metab Dispos. 2009, 37:1819-25.

15S Shi D, Yang D, Prinssen, Davies BE, Yan B. Surge in expression of carboxylesterase 1 during the post-neonatal stage enables a rapid gain of the capacity to activate the anti-influenza prodrug oseltamivir. The Journal of infectious diseases. 2011, 203:937-42

16S Frenneaux M, Stewart RA, Newman CM, Hallidie-Smith KA. Enalapril for severe heart failure in infancy. Arch Dis Child. 1989 64:219-23.

17S Leversha AM, Wilson NJ, Clarkson PM, Calder AL, Ramage MC, Neutze JM. Efficacy and dosage of enalapril in congenital and acquired heart disease. Arch Dis Child. 1994, 70:35-9.

18S Khalil F, Läer S. Development of a physiologically based model to support the choice of paediatric enalapril dosing regimen for orodispersible minitablets, 50th Annual Meeting of the AEPC, Rome June 1-4, 2016, P2-132.

Table S3: Dosing schedule for enalapril orodispersible minitablets

Age	Dose	Type of dose	Enalapril daily dose (ODMTs)	Enalapril daily dose (mg)	Enalapril morning dose	Enalapril evening dose
1 day to below 6 months (ca. 2.5 to 7 kg)	1st	Titration dose	10% of 1 ODMT 0.25 mg	0.025	10% of 1 ODMT 0.25 mg	-
	2nd	Titration dose	50% of 1 ODMT 0.25 mg	0.125	50% of 1 ODMT 0.25 mg	-
	3rd	Titration dose	1 ODMT 0.25 mg	0.25	1 ODMT 0.25 mg	-
	4th	Titration dose	2 ODMT 0.25 mg	0.5	1 ODMT 0.25 mg	1 ODMT 0.25 mg
	5th	Target dose	4 ODMT 0.25 mg	1	2 ODMT 0.25 mg	2 ODMT 0.25 mg
		Maximum dose	2 ODMT 1 mg	2	1 ODMT 1 mg	1 ODMT 1 mg
6 months to below 3 years (ca. 8 to 15 kg)	1st	Titration dose	1 ODMT 0.25 mg	0.25	1 ODMT 0.25 mg	-
	2nd	Titration dose	2 ODMT 0.25 mg	0.5	1 ODMT 0.25 mg	1 ODMT 0.25 mg
	3rd	Titration dose	4 ODMT 0.25 mg	1	2 ODMT 0.25 mg	2 ODMT 0.25 mg
	4th	Target dose	2 ODMT 1 mg	2	1 ODMT 1 mg	1 ODMT 1 mg
		Maximum dose	4 ODMT 1 mg	4	2 ODMT 1 mg	2 ODMT 1 mg
3 to below 8 years (ca. 16 to 25 kg)	1st	Titration dose	2 ODMT 0.25 mg	0.5	1 ODMT 0.25 mg	1 ODMT 0.25 mg
	2nd	Titration dose	4 ODMT 0.25 mg	1	2 ODMT 0.25 mg	2 ODMT 0.25 mg
	3rd	Titration dose	2 ODMT 1 mg	2	1 ODMT 1 mg	1 ODMT 1 mg
	4th	Target dose	4 ODMT 1 mg	4	2 ODMT 1 mg	2 ODMT 1 mg
		Maximum dose	8 ODMT 1 mg	8	4 ODMT 1 mg	4 ODMT 1 mg
8 to below 12 years (ca. 26 to 40 kg)	1st	Titration dose	4 ODMT 0.25 mg	1	2 ODMT 0.25 mg	2 ODMT 0.25 mg
	2nd	Titration dose	2 ODMT 1 mg	2	1 ODMT 1 mg	1 ODMT 1 mg
	3rd	Titration dose	4 ODMT 1 mg	4	2 ODMT 1 mg	2 ODMT 1 mg
	4th	Target dose	8 ODMT 1 mg	8	4 ODMT 1 mg	4 ODMT 1 mg
		Maximum dose	16 ODMT 1 mg	16	8 ODMT 1 mg	8 ODMT 1 mg

ODMT=orodispersible minitablets

Table S4: Results of ANOVA with group parameter AGE GROUP for enalapril and enalaprilat pharmacokinetic parameters maximum concentration (Cmax) and exposure (AUC)

PK parameter	Patients	df	Anova sum of squares	Mean square	F	p
ENA_AUCtau_ss_norm	all	2	0.29146947	0.14573473	0.30	0.7406
ENAAT_AUCtau_ss_norm	all	2	1.02611678	0.51305839	0.83	0.4406
ENA_Cmax_ss_norm	all	2	1.41021905	0.70510953	1.29	0.2802
ENAAT_Cmax_ss_norm	all	2	0.53131740	0.26565870	0.57	0.5690
tmax_ENA	all	2	6.76876446	3.38438223	3.35	0.0397
tmax_ENAAT	all	2	107.9805475	53.9902737	5.37	0.0064
ENA_AUCtau_ss_norm	DCM	2	0.50619295	0.25309647	1.80	0.1878
ENAAT_AUCtau_ss_norm	DCM	2	1.78308000	0.89154000	4.84	0.0181
ENA_Cmax_ss_norm	DCM	2	4.44974807	2.22487404	7.85	0.0025
ENAAT_Cmax_ss_norm	DCM	2	1.38258095	0.69129047	4.36	0.0254
tmax_ENA	DCM	2	5.53267543	2.76633771	3.30	0.0549
tmax_ENAAT	DCM	2	20.42161806	10.21080903	2.10	0.1448

ENA=enalapril, ENAAT=enalaprilat; AUCtau_ss_norm=exposure over dosing interval normalized for body weight and dose under steady state condition; Cmax_ss_norm= maximum concentration normalized for body weight and dose under steady state condition, DCM=Dilated Cardiomyopathy

Table S5: Pharmacokinetic parameters of enalapril and enalaprilat in naïve paediatric patients with heart failure due to DCM and CHD.

	n	Enalapril	Enalaprilat	Enalapril	Enalaprilat	Enalapril	Enalaprilat
		AUCtau,norm (ng/mL*h/mg*kg)		Cmax,norm (ng/mL/mg*kg)		tmax or tmax,ss (h)	
Total (All)	32	805.6 (0-3144.9	578.4 (0-3634.5)	266.9 (0-562.0)	79.4 (0-479.6)	2 (0-5.95)	6.05 (0-12.2)
1 day to <1 year (Ia)	30	824.7 (0-3144.9)	624.5 (0-3634.5)	266.9 (0-562.0)	83.1 (0-479.6)	2 (0-5.95)	6.05 (0-12.2)
1 year to <6 years (Ib)	2	437.2 (380.4-494.1)	270.1 (0-540.3)	217.1 (140.5-293.7)	36.6 (0-73.1)	1.5 (1-2)	6.01 (0-12.02)
Dilated Cardiomyopathy	3	494.1 (380.4-504.8)	260.4 (0-540.3)	140.5 (119.5-293.7)	55.8 (0-73.1)	1.98 (1-2)	6 (0-12.02)
1 months to <1 year (IIa)	1	504.8	260.4	119.5	55.8	1.98	6
1 year to <6 years (IIb)	2	437.2 (380.4-494.1)	270.1 (0-540.3)	217.1 (140.5-293.7)	36.6 (0-73.1)	1.5 (1-2)	6.01 (0-12.02)
Congenital Heart Disease	29	836.2 (0-3144.9)	632.5 (0-3634.5)	272.0 (0-562.0)	85.9 (0-479.6)	2 (0-5.95)	6.05 (0-12.2)
1 day to <1 year (IIIa)	29	836.2 (0-3144.9)	632.5 (0-3634.5)	272.0 (0-562.0)	85.9 (0-479.6)	2 (0-5.95)	6.05 (0-12.2)

Data reported as median (range), DCM=Dilated Cardiomyopathy; CHD=Congenital Heart Disease, AUCtau=Area under the curve within a dose interval of 12h; Cmax=maximal serum concentrations; tmax=time of maximal serum concentrations.

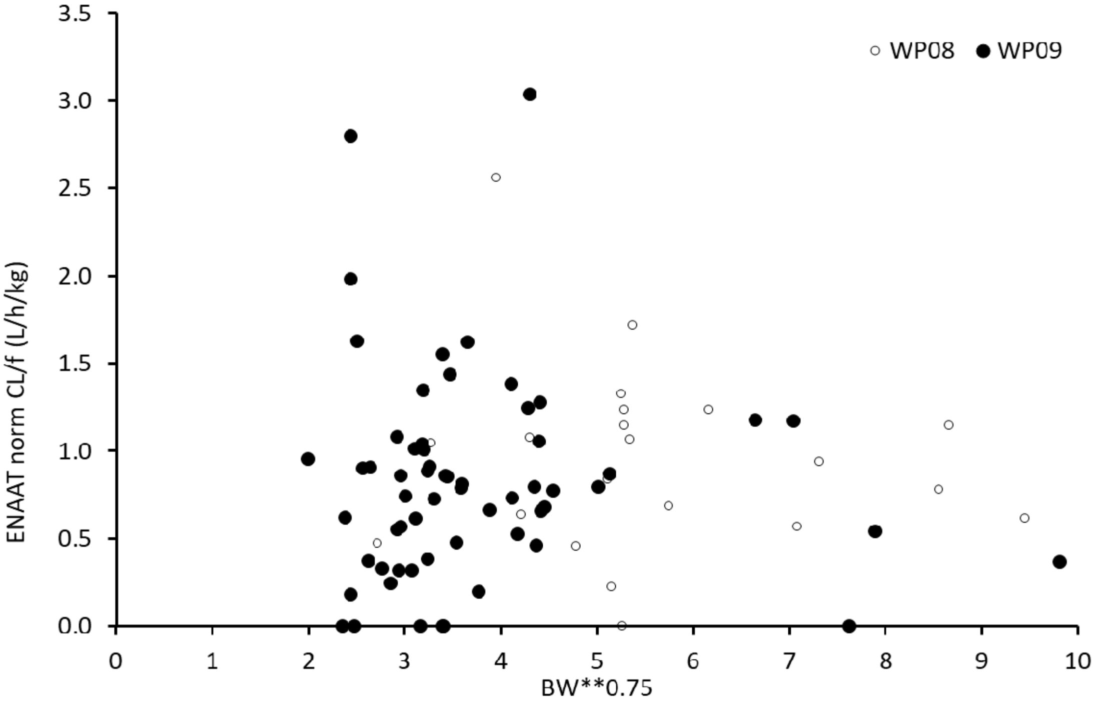

Figure S7: Allometric scaling of apparent oral clearance (normalized by body weight). of Enalaprilat (ENAAT) as function of age in patients with heart failure due to dilated cardiomyopathy (WP08 open circles, n=25) and due to congenital heart disease (WP09 closed circle, n=61).

Universitäres Herzzentrum Hamburg
Ein Unternehmen des UKE

lena

Enalapril Minischmelztabletten bei Kindern mit Herzinsuffizienz aufgrund einer dilatativen Kardiomyopathie

Liebe Eltern, Liebe/r Sorgeberechtigte/r!
Wir möchten Ihr Kind gerne einladen, an einer Studie mit einer neuen Darreichungsform von Enalapril in Form von Minischmelztabletten für Kinder teilzunehmen.

- Bevor Sie entscheiden, dass Ihr Kind an dieser Studie teilnehmen soll, ist es wichtig dass Sie verstehen, warum diese Studie durchgeführt wird und was es für Ihr Kind und Sie bedeutet teilzunehmen.
- Bitte nehmen Sie sich Zeit diese Patienteninformation genau durchzulesen. Besprechen Sie die Studie, wenn Ihr Kind alt genug ist, mit Ihrem Kind und wenn Sie möchten auch mit Freunden oder Verwandten.
- Es ist Ihre Entscheidung, ob Ihr Kind an dieser Studie teilnimmt oder nicht. Die Ablehnung der Teilnahme hat keine nachteiligen Folgen für die zukünftige medizinische Betreuung Ihres Kindes.
- Zögern Sie nicht Fragen zu stellen, wenn Sie etwas nicht verstehen oder wenn Sie genauere Informationen wünschen, bis Sie keine weiteren Fragen haben und Sie sicher sind, dass alles ausreichend beantwortet wurde.
- Nehmen Sie sich ausreichend Zeit um zu entscheiden, ob Ihr Kind teilnehmen soll oder nicht.
- Sie können die Teilnahme Ihres Kindes jederzeit ohne die Nennung von Gründen beenden.

Inhalt

1. Wichtige Informationen, die Sie zu Enalapril Minischmelztabletten haben sollten
2. Was ist der Zweck der klinischen Prüfung?
3. Enalapril Minischmelztabletten
4. Warum wird mein Kind gefragt teilzunehmen?
5. Wie läuft die klinische Prüfung ab?
6. Andere Behandlungs-möglichkeiten
7. Nutzen und Risiken
8. Kosten
9. Vorzeitige Beendigung und Widerruf der Einver-ständniserklärung
10. Wen kontaktiere ich im Fall von Problemen?
11. Vertraulichkeit der Daten
12. Weitere Informationen über die Studien-teilnahme
13. Kontaktdaten

Kontaktdaten

Für weitere Fragen im Zusammenhang mit dieser klinischen Prüfung stehen Ihnen Ihr Prüfarzt und seine Mitarbeiter gerne zur Verfügung.
Prof.Dr.med. Thomas Mir
Tel.: 040 / 74 10 - 5 37 18

1. Wichtige Informationen, die Sie zu Enalapril Minischmelztabletten haben sollten

- Enalapril ist für Erwachsene und Kinder eine wichtige und notwendige Behandlung für chronische Herzinsuffizienz. Es ist seit 1983 zugelassen und gehört zu den 10 meistverwendeten durch Schlucken verabreichten Medikamenten.
- Enalapril ist in Form von Tabletten und Kapseln zugelassen.
- Enalapril verhindert Blutgefäßverengungen, senkt den Blutdruck und verkleinert die Energiemenge, die für einen Herzschlag benötigt wird. Natürlich kann Enalapril auch Nebenwirkungen verursachen.
- Zurzeit ist Enalapril für Kinder unter 20 kg noch nicht offiziell zugelassen, auch wenn es häufig Kindern unter einem Jahr mit Herzinsuffizienz verschrieben wird. Das heißt, dass Enalapril als „off-label" Medikament an kleine Kinder verabreicht wird, weil es noch keine bessere Alternative gibt.
- Mit den neuen Minischmelztabletten soll die Verabreichung von Enalapril bei Kindern einfacher und zuverlässiger werden.

Es gibt bereits Daten, die die Wirkung von Enalapril bei Kindern bestätigen. Es gibt aber noch keine systematische Sammlung von Daten über die Sicherheit einer langzeitigen Verwendung (etwaige Nebenwirkungen) bei Kindern.

2. Was ist der Zweck der klinischen Prüfung

Über Jahre wurde bei Erwachsenen eine große Menge an Daten über die Sicherheit von Enalapril gesammelt. Obwohl Enalapril in der klinischen Routine häufig als Behandlung für Kinder mit Herzinsuffizienz verwendet wird, gibt es bis jetzt aber keine systematische und langzeitige Datensammlung mit einer an das Kindergewicht angepassten Dosis.
Ihr Kind wurde eingeladen, an der LENA Studie zu Pharmakokinetik und Pharmakodynamik von Enalapril teilzunehmen. Während dieser Studie wird Ihr Kind bis zu 8 Wochen lang Enalapril in Form von Minischmelztabletten bekommen.
Die Pharmakokinetik (PK) untersucht, wie der Körper das Medikament aufnimmt, verteilt und ausscheidet.
Die Pharmakodynamik (PD) untersucht wie das Medikament wirkt.

3. Enalapril Minischmelztabletten

In dieser Studie werden Enalapril Minischmelztabletten in 2 verschieden Stärken verwendet. Diese 2 Stärken sind in Ihrer Farbe eindeutig unterscheidbar: 1,0 mg Minischmelztabletten sind gelb, 0,25 mg Minischmelztabletten sind weiß.

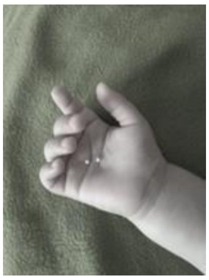

Der Prüfarzt wird die Behandlung Ihres Kindes mit sehr geringen Dosen beginnen und diese langsam bis zur optimalen Dosis für Ihr Kind steigern.
Ihr Kind soll die Enalapril Minischmelztabletten für 8 Wochen (56 Tage) ein- bis zweimal pro Tag nach Anweisung des Arztes einnehmen. Wenn Ihr Kind die Minischmelztabletten nicht mehr einnehmen soll, wird die Gesundheit Ihres Kindes dennoch für den Rest der 8 Wochen weiterhin überwacht. Die maximal empfohlene Dosis, die von Ihrem Kind eingenommen werden sollte, sind 8 Minischmelztabletten am Morgen und 8 Minischmelztabletten am Abend.

Die niedrigsten Dosen für Kinder unter 7 kg Körpergewicht werden nach Entscheidung des Arztes verdünnt. Diese verdünnten Dosen werden nur einmal am Tag verabreicht.

Der Prüfarzt oder eine qualifizierte Studienschwester wird Ihnen die Anwendung der Enalapril Minischmelztabletten genau erklären, bevor diese Ihrem Kind verabreicht werden. Sie bekommen die Minischmelztabletten für zuhause von Ihrem Prüfarzt oder einer qualifizierten Studienschwester.

<u>Wurde überprüft, ob diese Studie sicher ist?</u>
Die europäische Gesundheitsbehörde (EMA) hat bestätigt, dass es einen dringenden Bedarf an einer passenden und sicheren Verabreichungsform von Enalapril für Kinder gibt. Die unabhängige Ethikkommission der Landesärztekammer Hamburg hat diese Studie geprüft und positiv bewertet.

4. Warum wird mein Kind gefragt teilzunehmen?

Der Gesundheitszustand Ihres Kindes entspricht den Einschlusskriterien der LENA Studie. Dies und Ihre Einwilligung sind die Voraussetzungen für die Studienteilnahme.
Muss Ihr Kind an der Studie teilnehmen?
Es ist Ihre und die Entscheidung Ihres Kindes an der Studie teilzunehmen oder nicht. Ihr Prüfarzt wird Ihnen alle Einzelheiten erklären und mit Ihnen diese Patienteninformation besprechen. Wenn Sie entscheiden, dass Ihr Kind an dieser Studie teilnehmen soll, bitten wir Sie, eine Einverständniserklärung zu unterschreiben und den Anweisungen des Prüfarztes und dem Studienpersonal Folge zu leisten. Sie

können die Teilnahme an dieser Studie jederzeit ohne Nennung von Gründen beenden. Das hat keine negativen Auswirkungen auf die medizinische Behandlung Ihres Kindes.

5. Wie läuft die klinische Prüfung ab?

Wie ist die Studie organisiert?

Es werden insgesamt 50 Kinder in 5 Ländern (Österreich, Ungarn, Niederlande, Deutschland und Serbien) in 6 verschiedenen Krankenhäusern teilnehmen. Die Studie besteht aus einem Voruntersuchungsbesuch, unterschiedlich vielen Besuchen, um die optimale Dosis für Ihr Kind zu bestimmen, Studienkontrollbesuchen und einem Studienendbesuch.

Während der Studie sind die Krankenhausbesuche häufiger als normalerweise üblich. Dies ist notwendig um die Sicherheit Ihres Kindes zu gewährleisten und die optimale Dosis für Ihr Kind zu finden.

Abbildung 1: Studienablauf

Dilatative Kardiomyopathie Studienzeitpunkte in Tagen

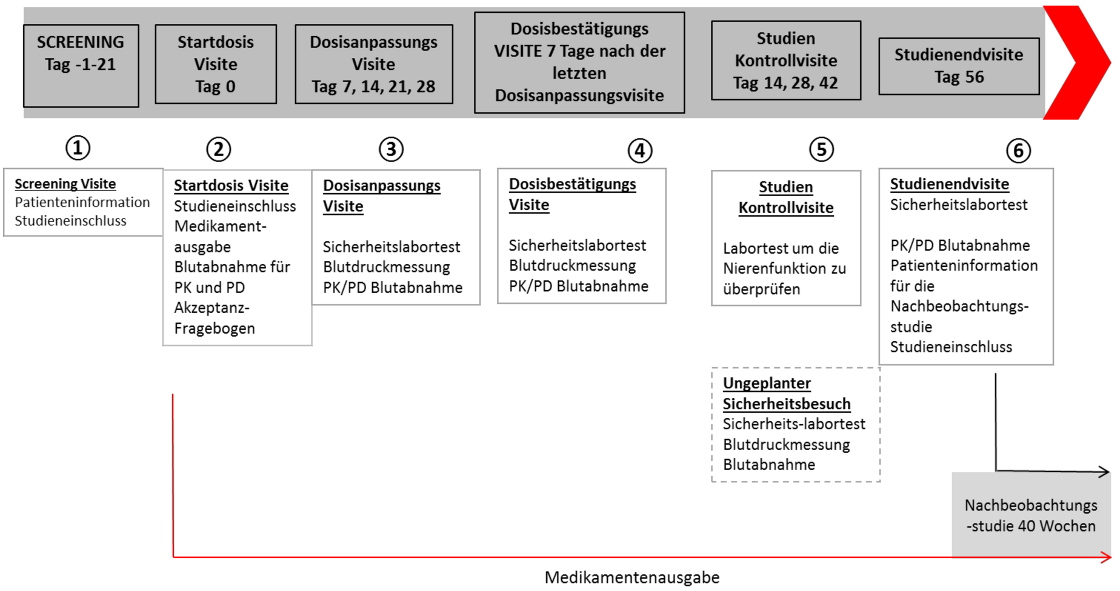

Folgende Untersuchungen und Tätigkeiten werden durchgeführt:

Was wird in dieser Studie untersucht?

Pharmakokinetische (PK) und pharmakodynamische (PD) Untersuchungen sollen helfen zu verstehen, wie die Minischmelztabletten im Körper funktionieren. Dafür wird

zu mehreren Zeitpunkten Blut abgenommen. Basierend auf den untersuchten Parametern wird die optimale Dosis für Ihr Kind verschrieben.
Das Wichtigste ist, dass alle unerwünschten Ereignisse Ihres Kindes während der 8 Wochen-Behandlung verständlich und vollständig gesammelt werden. Darum werden Sie gebeten, ein Tagebuch auszufüllen und dieses zu jedem Besuch mitzubringen. Außerdem wird die Nierenfunktion Ihres Kindes genau überwacht. Auch dafür muss bei jedem Besuch Blut abgenommen werden.
Weiter ist es wichtig, dass Sie Ihren Prüfarzt über alle Probleme, die beim Schlucken der Minischmelztabletten auftreten, informieren. Während der ersten Verabreichung der Minischmelztabletten wird ein Beobachtungstest zur Beurteilung der Akzeptanz und der Schmackhaftigkeit durchgeführt. Derselbe Test wird durchgeführt, wenn die optimale Dosis gefunden wurde und während des Studienendbesuchs. Bitte verabreichen Sie deshalb an diesen Tagen vor dem Krankenhausbesuch die Morgendosis nicht. Die Morgendosis wird an diesen Tagen von dem zuständigen Studienpersonal verabreicht, um die genaue Beobachtung durchzuführen.

Manche Kinder können eine stärkere Wirkung des Medikaments oder mehr Nebenwirkungen spüren als andere. Das kann mehrere Gründe haben wie das Alter Ihres Kindes oder die Art der Herzerkrankung. Ein anderer Grund kann auch die DNA Ihres Kindes, die genetische Zusammensetzung sein. Jeder Zelltyp hat seinen eigenen metabolischen „Fingerabdruck“, der die genaue Funktionsweise der Zelle erklären kann. Bei der Untersuchung des „Fingerabdruckes“ von Herzinsuffizienz aufgrund von dilatativer Kardiomyopathie sollen der Mechanismus und die Signalwege des menschlichen Körpers, die den Krankheitsgrad Ihres Kindes bestimmen, definiert werden. Außerdem soll untersucht werden, wie der “Fingerabdruck“ von Enalapril beeinflusst werden kann.
Diese Parameter sind Thema von separaten Unter-Studien. In den Unter-Studien wird untersucht, ob und in welchem Ausmaß genetische und metabolische Faktoren die Wirkung der Behandlung von Herzinsuffizienz aufgrund von dilatativer Kardiomyopathie im Allgemeinen und im speziellen Fall einer Behandlung mit Enalapril, beeinflussen. Sie werden eine eigene Patienteninformation und Einverständniserklärung zu den Unter-Studien erhalten und können unabhängig entscheiden, ob Ihr Kind auch an diesen Unter-Studien teilnehmen soll.

Was müssen Sie und Ihr Kind machen?

Während des ersten Besuchs, dem Voruntersuchungsbesuch, wird Ihr Kind untersucht, ob es an der Studie teilnehmen kann. Wenn ja, werden Sie gebeten, regelmäßig in das Krankenhaus zu kommen. Während der Krankenhausbesuche werden verschiedene Untersuchungen durchgeführt um sicher zu stellen, dass die optimale Dosis für Ihr Kind erreicht wird. Es werden bei jedem Besuch Blut- und Urinproben genommen, um die Nierenfunktion Ihres Kindes zu überwachen.
Zuhause müssen Sie Ihrem Kind täglich die vom Prüfarzt verschriebenen Minischmelztabletten verabreichen. Sie werden gebeten, alle nicht verwendeten Enalapril Minischmelztabletten sowie die leeren Dosen beim nächsten Besuch ins

Krankenhaus zurückzubringen.

Beschreibung der Tätigkeiten an den einzelnen Besuchen (die eingekreisten Zahlen beziehen sich auf Abbildung 1 auf Seite 4 dieser Patienteninformation.)

- Voruntersuchungsbesuch①

Vor oder beim ersten Studienbesuch wird Sie Ihr Arzt über die Studie genau informieren, Ihnen alle Bedingungen für die Studie genau erklären und Ihnen alle Fragen beantworten. Danach werden Sie gebeten, die Einverständniserklärung zu unterschreiben. Wenn Ihr Kind alt genug ist, wird es auch über die Studie aufgeklärt und kann auch eine eigene Einverständniserklärung für Kinder unterschreiben. Außerdem wird während dieses Besuches Blut abgenommen und eine Urinprobe genommen um festzustellen, ob ihr Kind für die Teilnahme an der Studie geeignet ist. Das Herz wird mit EKG und Echo untersucht. Ihr Arzt wird Ihnen einige Fragen über die Krankengeschichte Ihres Kindes stellen. Wenn Ihr Kind an der Studie teilnehmen kann, bekommen Sie eine Patientenkarte, die über die Teilnahme Ihres Kindes an dieser Studie informiert, falls Sie während der Studienteilnahme einen anderen Arzt kontaktieren.

Sie werden außerdem einen Termin für den nächsten Besuch bekommen.

- Erster Dosis Besuch②

Bei diesem Besuch wird Ihr Kind zum ersten Mal Minischmelztabletten bekommen. Danach wird der Blutdruck über 8 Stunden alle 30 Minuten gemessen. Bitte bringen Sie einen Laptop, ein Buch oder ein Spielzeug ins Krankenhaus mit. Außerdem werden während dieses Besuchs auch mehrere Blutproben und eine Urinprobe genommen. Zum Schluss erhalten Sie von Ihrem Arzt Enalapril Minischmelztabletten und Sie können das Krankenhaus verlassen. Falls Ihr Kind stationär aufgenommen ist, werden die Minischmelztabletten im Krankenhaus verabreicht.

Sie erhalten ein Patiententagebuch, um unerwünscht aufgetretene Ereignisse, die Ihnen bei Ihrem Kind auffallen, zu notieren. Außerdem schreiben Sie bitte die Zeit der Verabreichung der Minischmelztabletten an den letzten 7 Tagen vor jedem weiteren Krankenhausbesuch auf.

Der Besuch ① und ② kann auch an einem Tag gemeinsam erfolgen. Diese Entscheidung wird von Ihrem Arzt aufgrund des Gesundheitszustands Ihres Kindes getroffen.

- Titrationsbesuch③

Sie werden gebeten, bis zu 5 Mal ins Krankenhaus zu kommen, um die Dosis zu steigern und die optimale Dosis für Ihr Kind zu finden. Während diesen Besuchen wird der Blutdruck Ihres Kindes gemessen, Blutproben sowie eine Urinprobe werden genommen und die Studienschwester wird das Patiententagebuch mit Ihnen

durchspechen. Sie werden bei jedem Besuch neue Minischmelztabletten in einer neuen Dosierung von Ihrem Arzt erhalten.

BITTE: Bringen Sie bei jedem Besuch alle Enalapril Minischmelztablettendosen und das Patiententagebuch mit.

- Wenn die optimale Dosis gefunden wurde, wird diese in einem "Dosis-Bestätigungsbesuchs"④ bestätigt, in dem noch einmal dieselben Untersuchungen durchgeführt werden. Danach werden Sie gebeten alle 2 Wochen zu regelmäßigen Studienkontrollbesuchen zu kommen.
- Bei diesen Studienkontrollbesuchen⑤ wird Ihr Kind körperlich untersucht und gewogen. Desweiteren werden der Blutdruck und der Puls gemessen sowie eine Blut-und Urinprobe genommen. Die Studienschwester wird mit Ihnen gemeinsam das Patiententagebuch überprüfen. Der Prüfarzt wird Sie über aufgetretene Erkrankungen und eingenommene Medikamente seit dem letzten Besuch befragen. Die Einnahme der Enalapril Minischmelztabletten wird vom Studienpersonal beobachtet und dokumentiert. Zum Schluss erhalten Sie neue Minischmelztabletten für Ihr Kind sowie das Patiententagebuch.

- Studienendbesuch nach 8 Wochen (56 Tage)⑥

BITTE: Bringen Sie bei jedem Besuch alle Enalapril Minischmelztablettendosen und das Patiententagebuch mit.

Bei diesem Besuch wird eine komplette Untersuchung der Gesundheit Ihres Kindes durchgeführt:

Zusätzlich zu der körperlichen Untersuchung, der Messung von Körpergröße und –gewicht, Blutdruck und Puls, der Entnahme einer Blut- und Urinprobe, der Beobachtung der letzten Enalapril Minischmelztabletteneinnahme und der Überprüfung des Tagebuchs auf unerwünschte Ereignisse, zwischenzeitlich aufgetretene Erkrankungen und Begleitmedikamente werden ein EKG und eine Echokardiographie durchgeführt.

Wiederholte Blutabnahmen erfolgen nur einmal während der Studie. Ihr Arzt entscheidet, wann, während welchen Besuches, diese Blutabnahmen gemacht werden. Bei allen anderen Besuchen wird Ihrem Kind nur einmal Blut abgenommen.

Wenn Sie die Studienteilnahme Ihres Kindes vor Ablauf der 8 Wochen beenden wollen, machen Sie bitte mit Ihrem Arzt einen Studienendbesuch aus.

Wenn Ihr Kind eine Operation benötigt, wird die Einnahme der Enalapril Minischmelztabletten für eine gewisse Zeit gestoppt und, wenn Ihr Arzt Ihr Kind für gesundheitlich stabil erklärt und Sie weiterhin einwilligen, wieder gestartet. Eine Operation kann zu jeder Zeit während der Studie durchgeführt werden.

Blut- und Urinproben
Die Blutmenge, die während dieser Studie Ihrem Kind abgenommen wird, hängt vom Gewicht Ihres Kindes und vom Zeitpunkt der wiederholten Blutabnahme ab.
Während der Studie, vom ersten bis zum letzten Besuch, werden von Ihrem Kind, wenn es ein Gewicht von unter 3,0kg hat, maximal 12,5ml Blut abgenommen. Wenn Ihr Kind in der Unter-Studie teilnimmt, werden insgesamt 14,0ml Blut abgenommen. Bei jedem einzelnen Besuch werden nicht mehr als 3,3ml Blut abgenommen.
Wenn Ihr Kind zwischen 3,0 - 3,6kg wiegt, werden insgesamt 14,0ml Blut (ohne Unter-Studie) und mit der Unter-Studie 15,5ml Blut abgenommen. Wenn Ihr Kind über 3,6kg wiegt, werden insgesamt 15,1ml Blut (ohne Unter-Studie) und mit der Unter-Studie 17,6ml Blut abgenommen.
Bei jedem einzelnen Besuch werden nicht mehr als 3,8ml Blut abgenommen. Das Blut wird mittels Nadelstich in eine Vene entnommen.
Bei jedem Besuch in der Studie wird eine Urinprobe (wenige Tropfen sind ausreichend) genommen, um die Nierenfunktion zu überprüfen.

Begleitmedikamente
Ihr Kind darf während dieser Studie alle weiteren Medikamente, die ihm von Ihrem Prüfarzt verschrieben werden, einnehmen. Wenn Ihr Kind zusätzliche Medikamente benötigt, fragen Sie bitte zuerst Ihren Prüfarzt, ob diese mit Enalapril Wechselwirkungen haben könnten. Bei jedem Besuch im Krankenhaus werden Sie nach allen Medikamenten Ihres Kindes gefragt.

Einschränkungen
Es gibt keine Beschränkungen in der Ernährung und auch sonst keine Einschränkungen. Um Verschlucken zu verhindern, kann Ihr Kind Wasser oder Milch zum Schlucken der Minischmelztabletten trinken.

Welche Therapiemöglichkeit gibt es nach der Teilnahme an dieser Studie?
Nach der Studienteilnahme mit 8 Wochen Behandlung ist es möglich, die Behandlung mit Enalapril Minischmelztabletten bis zu 9 Monate innerhalb einer Nachbeobachtungsstudie fortzusetzen, jedoch maximal bis zum 31.Juli, 2018. Wenn Ihr Kind danach noch Enalapril benötigt, oder wenn Sie nicht möchten, dass Ihr Kind an der Nachbeobachtungsstudie teilnimmt, wird Ihr Arzt Ihnen am Markt verfügbare Enalapril Tabletten in einer Apothekenzubereitung für Kinder (Kapseln) verschreiben, wie sie derzeit routinemäßig verwendet werden.

6. Was gibt es für andere Behandlungsmöglichkeiten?

Wenn Sie nicht wollen, dass Ihr Kind an dieser Studie teilnimmt, wird Ihr Arzt Ihrem Kind Enalapril oder Captopril, oder einen anderen ACE-Hemmer, verschreiben. Diese werden routinemäßig für die Behandlung von Kindern mit dilatativer Kardiomyopathie eingesetzt. Sie werden aber nur als Tabletten für größere Kinder und Erwachsene vermarktet und müssen daher in der Apotheke in für kleinere Kinder geeigneten

Darreichungsformen zubereitet werden. Captopril hat außerdem einen stärkeren Effekt auf den Blutdruck als Enalapril und ist deswegen nicht so gut für die Behandlung von Kindern geeignet.

7. Nutzen und Risiken

Was sind der Nutzen bzw. die Risiken der Teilnahme?

<u>Möglicher Nutzen:</u>
Enalapril in Form von Minischmelztabletten zu verabreichen macht es für Sie und Ihr Kind einfacher und sicherer die benötigte Dosis einzunehmen. Die Tabletten müssen nicht mehr zerbrochen und verdünnt werden. Sie können also sicher sein, dass Ihr Kind genau die Dosis bekommt, die Ihr Arzt in der momentanen Situation für die richtige hält.
Studien in mehr als 500 Kindern zeigten, dass Minischmelztabletten von Kindern deutlich besser akzeptiert werden als Saft.

Während der Teilnahme an dieser Studie wird die Gesundheit Ihres Kindes genauer überwacht als normalerweise.
Außerdem können Sie mit der Aufzeichnung der Sicherheitsdaten von Ihrem Kind helfen, dass andere Kinder mit Herzinsuffizienz in Zukunft eine bessere Behandlung erhalten werden.

<u>Mögliche Risiken und Belastungen:</u>

- Durch die Teilnahme an dieser Studie müssen Sie und Ihr Kind das Krankenhaus öfter besuchen als normalerweise.

- Auch wenn die Menge des abgenommenen Blutes kein Problem für Ihr Kind ist und von kinderheilkundlichen klinischen Richtlinien erlaubt ist, wird es für Ihr Kind mehr Blutabnahmen als während der normalen klinischen Praxis geben.

- Blutabnahmen sind mit folgenden Risiken verbunden: in 1 – 3% der Fälle blaue Flecken, lokale Irritationen mit juckender Haut, kleine Blutungen und Entzündungen. In seltenen Fällen (in weniger als in 1 von 10 000 Blutabnahmen) kann es zu Thrombosen (Verstopfung der Blutgefäße), einer Infektion oder einer Nervenschädigung kommen. Manche Kinder fühlen sich auch geschwächt aufgrund des lokalen Schmerzes.

- Die Einnahme von Enalapril kann nicht nur die gewünschten Wirkungen auf das Herz Ihres Kindes, sondern auch unerwünschte Wirkungen haben. Die Nebenwirkungen in Kindern dürften ähnlich den Nebenwirkungen in Erwachsenen mit Herzinsuffizienz sein: in 1% bis 10% der Fälle kann es zu verschwommenem Sehen und Schwindel, Kopfschmerzen, niedrigem Blutdruck, Husten, Depression oder allergischen Reaktionen wie Schwellung des Gesichtes, der Lippen, der

Zunge oder des Halses mit Schluckbeschwerden, zu Atembeschwerden oder erhöhten Kaliumwerten und Kreatininwerten im Blut kommen. Deutlich seltener treten Müdigkeit, Muskelschwäche, Bauch- und Brustschmerzen, Übelkeit, Erbrechen oder Durchfall auf. Bei einigen wenigen Kindern wurden Bronchitis, Lungenentzündung und Harnwegsinfektionen oder eine Änderung des Geschmackssinns gesehen.

- Seltene Nebenwirkungen, die bei Erwachsenen beobachtet wurden sind: plötzlicher Blutdruckabfall, schneller bzw. unregelmäßiger Herzschlag (Palpitationen), Herzinfarkt (möglich durch sehr niedrigen Blutdruck in Hochrisikopatienten wie Patienten mit Durchblutungsproblemen des Herzens oder Gehirns), Anämie (einschließlich aplastischer und hämolytischer), Schlaganfall (möglich durch sehr niedrigen Blutdruck in Hochrisikopatienten), Verwirrung, Schlaflosigkeit oder Schläfrigkeit, Nervosität, prickelndes Gefühl in der Haut oder Taubheitsgefühl der Haut, Schwindel, Läuten in den Ohren (Tinnitus), laufende Nase, Halsentzündung oder Heiserkeit, Asthma, langsame Nahrungsbewegung durch den Dünndarm, Entzündung der Bauchspeicheldrüse, Erbrechen, Magenverstimmung, Verstopfung, Appetitlosigkeit, gereizter Magen, trockener Mund, Magengeschwür, geschwächte Nierenfunktion, verstärktes Schwitzen, juckender Ausschlag oder Nesselausschlag, Haarverlust, Muskelkrämpfe, Hautrötung, Unwohlgefühl, erhöhte Temperatur (Fieber), Impotenz, erhöhter Proteinspiegel im Harn, verringerter Blutzuckerspiegel, verringerter Natriumwert im Blut, erhöhter Harnstoffwert im Blut.

- In frühgeborenen Patienten kann Enalapril Schädelunterentwicklung, Harnverhalt, niedrigen Blutdruck, Nierenversagen und Tod hervorrufen.

- Wie bei jedem Medikament können auch neue, nicht bekannte und nicht vorhersehbare Nebenwirkungen auftreten.

Sie werden über jede neue Information, Erkenntnis sowie Änderung der Studie, die Ihr Einverständnis zur Teilnahme beeinflussen könnte, mündlich informiert.

Bei jedem Krankenhausbesuch werden Sie gebeten alle (ungewöhnlichen) Ereignisse des Gesundheitszustandes Ihres Kindes mitzuteilen.

8. Kosten

Sie werden durch die Teilnahme Ihres Kindes an dieser Studie keinerlei finanzielle Belastungen haben. Sie müssen die Medikamente und Untersuchungen der Studie nicht bezahlen.

Etwaige Anreisekosten werden auf Basis vorgelegter Zahlungsnachweise erstattet. In begründeten Fällen können Sie für Studientage Aufwandsentschädigungen bis zu einer maximalen Gesamthöhe von € 300.00 in Ihrem Prüfzentrum beantragen.

9. Frühzeitige Beendigung und Widerruf der Einverständniserklärung

Sie können Ihr Einverständnis zur Teilnahme Ihres Kindes an der klinischen Studie ohne Angabe von Gründen jederzeit zurückziehen. Die Entscheidung, dass Ihr Kind frühzeitig die Teilnahme an der Studie beendet, ist mit keinen Nachteilen für Ihr Kind verbunden. In diesem Fall werden keine zusätzlichen Daten von Ihrem Kind gesammelt und vorliegende noch nicht analysierte biologische Proben werden umgehend zerstört. Ergebnisse von Proben, die zum Zeitpunkt des Entzugs der Teilnahme bereits analysiert waren, werden jedoch zu weiteren Studienzwecken verwendet. Auch andere studien-relevante Daten die erforderlich sind um sicherzustellen, dass die Wirkung und Sicherheit der Enalapril Minischmelztabletten auch durch die für eine Marktzulassung zuständigen Behörden beurteilt werden können, und dass die schutzwürdigen Interessen Ihres Kindes nicht beeinträchtigt werden, werden aufbewahrt.

Es ist aber auch möglich, dass Ihr Studienarzt (oder gegebenenfalls der Auftraggeber dieser klinischen Prüfung) entscheidet, die Teilnahme Ihres Kindes an der klinischen Prüfung vorzeitig zu beenden, ohne vorher Ihr Einverständnis einzuholen. Die Gründe hierfür können sein:

a) Ihr Kind könnte den Erfordernissen der Klinischen Prüfung nicht mehr entsprechen;

b) Ihr Studienarzt hat den Eindruck, dass eine weitere Teilnahme an der klinischen Prüfung nicht in Ihrem Interesse ist, z.B., wenn er feststellt, dass die Anreise zu den Studienterminen eine zu große Belastung für Ihr Kind bedeutet;

c) Der Auftraggeber trifft die Entscheidung, die gesamte klinische Prüfung abzubrechen. Dies könnte eintreffen, wenn neue Erkenntisse aus anderen Studien Zweifel an der Eignung von Enalapril bei Kindern aufkommen lassen sollten.

Sofern Sie sich dazu entschließen, dass Ihr Kind vorzeitig aus der klinischen Prüfung ausscheidet, oder seine Teilnahme aus einem der oben genannten Gründe vorzeitig beendet wird, ist es wichtig, dass Sie sich einen Studienendbesuch ausmachen. Dabei wird Ihr Kind noch einmal gründlich untersucht und Sie werden gebeten die Studienmedikamente zurückzubringen.

10 Wen kann ich im Fall von Problemen kontaktieren?

Bei auftretenden Problemen oder Fragen können Sie sich jederzeit an die betreffenden, mit der Studie betrauten Personen wenden, nähere Informationen und Kontaktdaten finden Sie unter Punkt 13.

11. Datenschutz und Vertraulichkeit

Der Auftraggeber der Studie (Ethicare GmbH, Deutschland) trifft alle möglichen

Maßnahmen, um die Identität Ihres Kindes zu schützen. Die Prüfärzte und ihre Mitarbeiter unterliegen im Umgang mit den Daten den Bestimmungen des deutschen Arzneimittelgesetzes in der jeweils geltenden Fassung. Ausschließlich autorisiertes Studienpersonal - wie Mitglieder des Studienteams am Prüfzentrum, Studienmonitore des Auftraggebers sowie Mitglieder der zuständigen Behörde - hat Einblick in die personenbezogenen Daten Ihres Kindes, in denen es namentlich genannt wird. Personenbezogene Daten wie das Geburtsdatum oder der Name Ihres Kindes werden durch eine spezielle Studiennummer ersetzt und dadurch pseudonymisiert. Nur das Studienteam und der behandelnde Arzt am Prüfzentrum wissen, welche Studiennummer Ihr Kind hat. Die pseudonymisierten Daten Ihres Kindes werden zur weiteren Auswertung an die Firma Ethicare oder an ein, durch die Firma Ethicare autorisiertes Institut gesendet. Die im Rahmen eines Reports oder einer Publikation veröffentlichen Studiendaten lassen keine Rückschlüsse auf die Identität Ihres Kindes zu.

12. Weitere Informationen zur Studienteilnahme

<u>Wer organisiert und finanziert die klinische Studie?</u>

Die Studie ist Teil des von der Europäischen Kommission finanzierten „LENA Projektes“ unter der Leitung der Universität Düsseldorf. Der Auftraggeber der Studie ist die Firma Ethicare GmbH mit Sitz in Haltern am See, Deutschland. Ihr Prüfarzt erhält für die Einbringung von Patienten in diese Studie einen Anteil der öffentlichen Förderung.

<u>Was geschieht, wenn neue Informationen über Enalapril Minischmelztabletten bekannt werden?</u>

Ihr Prüfarzt wird Sie über alle neuen Erkenntnisse, die in Bezug auf diese klinische Prüfung bekannt werden, und für Sie wesentlich werden könnten, umgehend informieren. Auf dieser Basis können Sie dann Ihre Entscheidung zur weiteren Teilnahme an dieser klinischen Prüfung neu überdenken.

<u>Was geschieht mit den biologischen Proben unseres Kindes?</u>

Die gesammelten Blut- und Urinproben werden, wie die persönlichen Daten Ihres Kindes, mit einer speziellen Studiennummer versehen, und entweder im Krankenhaus vor Ort untersucht oder zur weiteren Analyse nach Düsseldorf gesendet. Nach Studienende und nach erfolgreicher Medikamentenzulassung werden alle verbleibenden biologischen Proben zerstört.
Sie können den verantwortlichen Arzt nach Studienende kontaktieren und ihn nach den über Ihr Kind gesammelten Gesundheitsdaten fragen.

<u>Werden in dieser Studie genetische Tests durchgeführt?</u>

Ihr Kind kann entsprechend Ihrer Entscheidung an sogenannten Unter-Studien teilnehmen. In diesen Unter-Studien werden die entnommenen Blutproben Ihres Kindes genetischen und metabolischen Tests unterzogen. Weitere Informationen zur

Durchführung der Unter-Studien finden Sie in der separaten Patienteninformation.

Was geschieht mit den Ergebnissen der Studie?

Studienergebnisse sowie weitere studienspezifische Informationen werden nach Beendigung der Studie sowohl an die zuständige Ethikkommission als auch an die Behörde weitergeleitet, in den jeweiligen öffentlichen Datenbanken gespeichert und in Fachzeitschriftern veröffentlicht werden.

Wer hat den Inhalt der Studie und deren Durchführung genehmigt?

Die Fragestellung sowie die Abläufe zur Durchführung dieser Studie wurden von der Ethikkommission der Landesärztekammer Hamburg begutachtet und positiv bewertet und von der zuständigen deutschen Behörde, dem Bundesinstitut für Arzneimittel und Medizinprodukte (BfArM), begutachtet und genehmigt.

Möglichkeiten zur Diskussion und weitere Fragen

Für weitere Fragen im Zusammenhang mit dieser klinischen Studie stehen Ihnen Ihr Studienarzt und seine Mitarbeiter gerne zur Verfügung. Auch Fragen, die Ihre Rechte als Eltern/Sorgeberechtigter des Patienten und Teilnehmer an dieser klinischen Prüfung betreffen, werden Ihnen gerne beantwortet.

Versicherungsschutz

Als Teilnehmer an dieser klinischen Prüfung besteht für Ihr Kind der gesetzlich vorgeschriebene verschuldensunabhängige Haftpflichtversicherungsschutz. Dieser deckt alle Schäden ab, die am Leben Ihres Kindes oder seiner Gesundheit durch die an ihm/ihr durchgeführten Maßnahmen der klinischen Prüfung verursacht werden können, mit Ausnahme von Schäden auf Grund von Veränderungen des Erbmaterials in Zellen der Keimbahn. Unfälle, die auf dem Weg zur oder von der Klinik nach Hause geschehen, werden für Ihr Kind und Sie von einer Wege-Unfallversicherung abgedeckt.

Beide Versicherungen wurden abgeschlossen bei

Name und Anschrift der Versicherung:	ACE European Group Limited<br>A Chubb Company<br>Direktion für Deutschland<br>Lurgiallee 12<br>60439 Frankfurt am Main
Telefon:	069 75613 0
Fax:	069 746193
Versicherungsnummer:	DECANA16190

Deckungssumme der Haftpflichtversicherung: € 500.000,-- je Patient Deckungssumme der Wege-Unfallversicherung, die auch die Begleitperson(en)

schützt: € 100.000,- bei Invalidität oder Tod

Eine Beschreibung der Versicherungsbedingungen liegt dieser Patientenaufklärung bei.

Im Schadensfall können Sie sich direkt an den Versicherer wenden und Ihre Ansprüche selbständig geltend machen. Für den Versicherungsvertrag ist deutsches Recht anwendbar, die Versicherungsansprüche sind in Deutschland einklagbar.

Um den Versicherungsschutz nicht zu gefährden

- dürfen Sie sich während der Dauer der klinischen Prüfung einer anderen medizinischen Behandlung nur im Einvernehmen mit Ihrem behandelnden Prüfarzt unterziehen (ausgenommen davon sind Notfälle). Dies gilt auch für die zusätzliche Einnahme von Medikamenten oder die Teilnahme an einer anderen Studie.
- müssen Sie dem behandelnden Prüfarzt - oder der oben genannten Versicherungsgesellschaft - eine Gesundheitsschädigung, die als Folge der klinischen Prüfung eingetreten sein könnte, unverzüglich mitteilen.
- müssen Sie alles Zumutbare tun um Ursache, Hergang und Folgen des Versicherungsfalles aufzuklären und den entstandenen Schaden gering zu halten. Dazu gehört ggf. auch, dass Sie Ihre behandelnden Ärzte ermächtigen, vom Versicherer geforderte Auskünfte zu erteilen.

13. Kontaktdaten

Die Gesundheit und das Wohlbefinden Ihres Kindes hat für uns oberste Priorität. Ein mit der Studie betrauter Arzt steht Ihnen für mögliche Rückfragen gerne zur Verfügung. Sollten im Rahmen der Studie Probleme z.B. in Form von Nebenwirkungen auftreten, können Sie sich unter den unten angeführten Kontaktdaten jederzeit an Ihren Prüfarzt oder an einen mit der Studie betrauten Arzt wenden:

Prof. Dr.med. Thomas Mir

Tel.: 040 / 74 10 - 5 37 18
Universitätsklinikum Hamburg Eppendorf, UKE,
Universitäres Herzzentrum,
Klinik für Kinderkardiologie / Herzchirurgie für angeborene Herzfehler
(Auf dem Gelände des UKE: Gebäude O70 Gebäude N23)
Martinistraße 52
20246 Hamburg

Es existiert außerdem eine Kontaktstelle bei der zuständigen Bundesoberbehörde.
Teilnehmer an klinischen Prüfungen, ihre gesetzlichen Vertreter oder

Sorgeberechtigte können sich an diese Kontaktstelle wenden:

Bundesinstitut für Arzneimittel und Medizinprodukte
Fachgebiet Klinische Prüfung / Inspektionen
Kurt-Georg-Kiesinger-Allee 3
53175 Bonn
Telefon: 0228 / 207-4318 Fax: 0228 / 207-4355 e-mail: ct@bfarm.de

Study Centre:
Study Title:
Study Number: 2015-602295-01
EudraCT Number: 2015-002335-17

Informed Consent Form for the participation of your child in a clinical study

____

Patient name (BLOCK LETTERS)

____

Patient Screening number

I and my child have been informed by his/her responsible study physician or deputy study physician or qualified study personnel about the nature, scope and significance of the above mentioned clinical study.

____

Name of study physician (BLOCK LETTERS)

1. I confirm that I have read and understand the information sheet dated …………(Version…..) for the above study. I have had the opportunity to consider the information, ask questions and have had these answered satisfactorily.
2. I understand that my child´s participation is voluntary and that I am free to withdraw at any time without giving a reason, without my child´s medical care or legal rights being affected.
3. The procedures and conduct of the study have been explained to me. I have been informed about possible benefits, risks (side effects) and burden of participation. Furthermore, I have been informed about my and my child´s obligations with regard to cooperation with the study physician and personnel at the study centre, especially about the obligation to inform my child´s study physician immediately about any change in the condition of my child´s health.
4. I agree that authorised representatives from the LENA project or representatives from regulatory authorities or ethics committees may review my child´s personal data or my child´s pseudonymised data, collected in the course of the study might be transmitted to Ethicare and to national and international regulatory authorities for review. The inspection of the data serves to assure the quality and integrity of the data as required by regulatory requirements.
5. I further consent to the pseudonymised processing and storage of my data collected during the course of the study and to transmission of such data to Ethicare or to an institute authorised by Ethicare. This is necessary for the subsequent statistical analysis of the study data.
6. I have been informed that my child´s personal data will be treated with strict confidentiality and will not become accessible to the general public.
7. I agree on my child taking part in the above study.

I received a copy of the signed and dated written information and consent for participation in this study.

Name of the person acting on behalf of the patient	Date	Signature
Name of the person acting on behalf of the patient	Date	Signature
Name of the person taking consent	Date	Signature

ABOUT THE LENA PROJECT

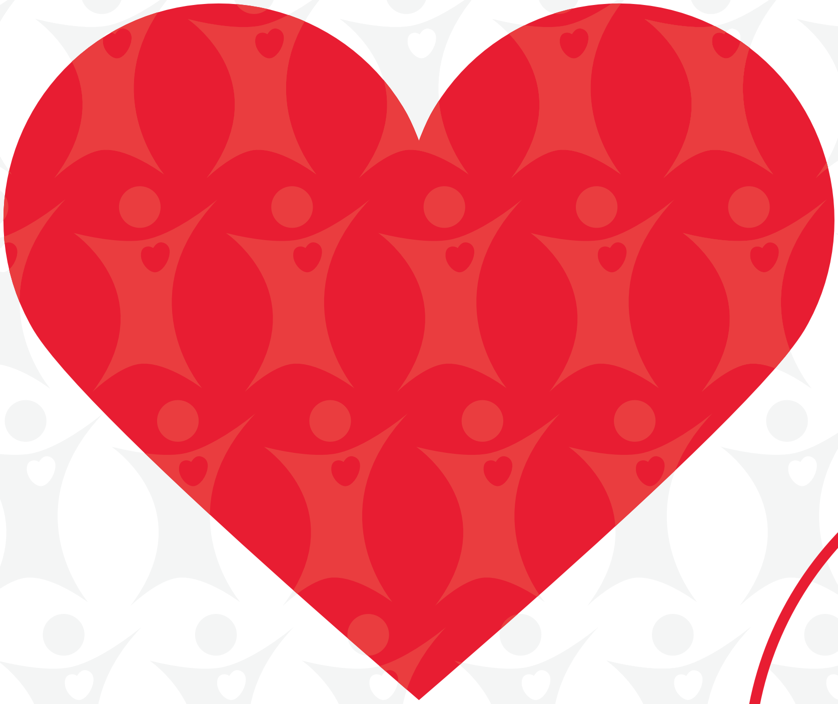

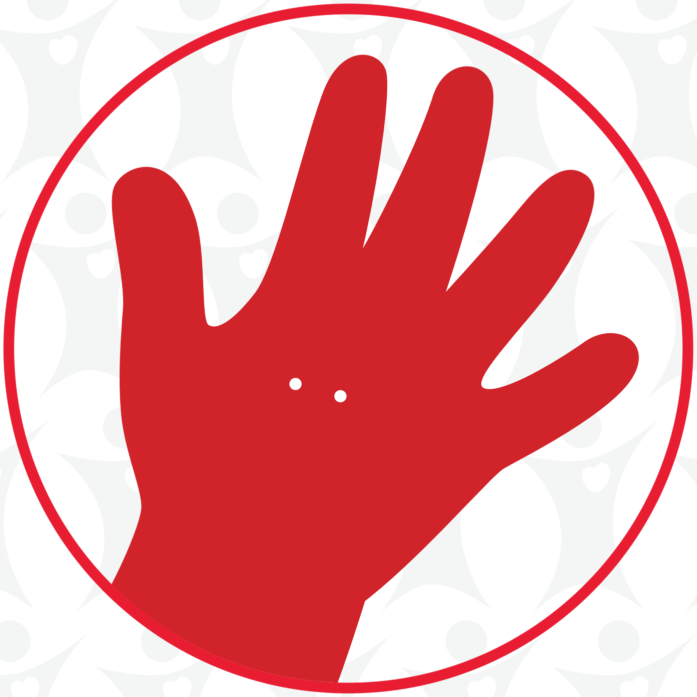

new mini-tablets
to help poorly hearts

Assent Form A | 0-5 Years | Version 1 | 03 Aug 15 | Protocol No: 2015-602295-01 / 2015-602295-02

DOCTOR

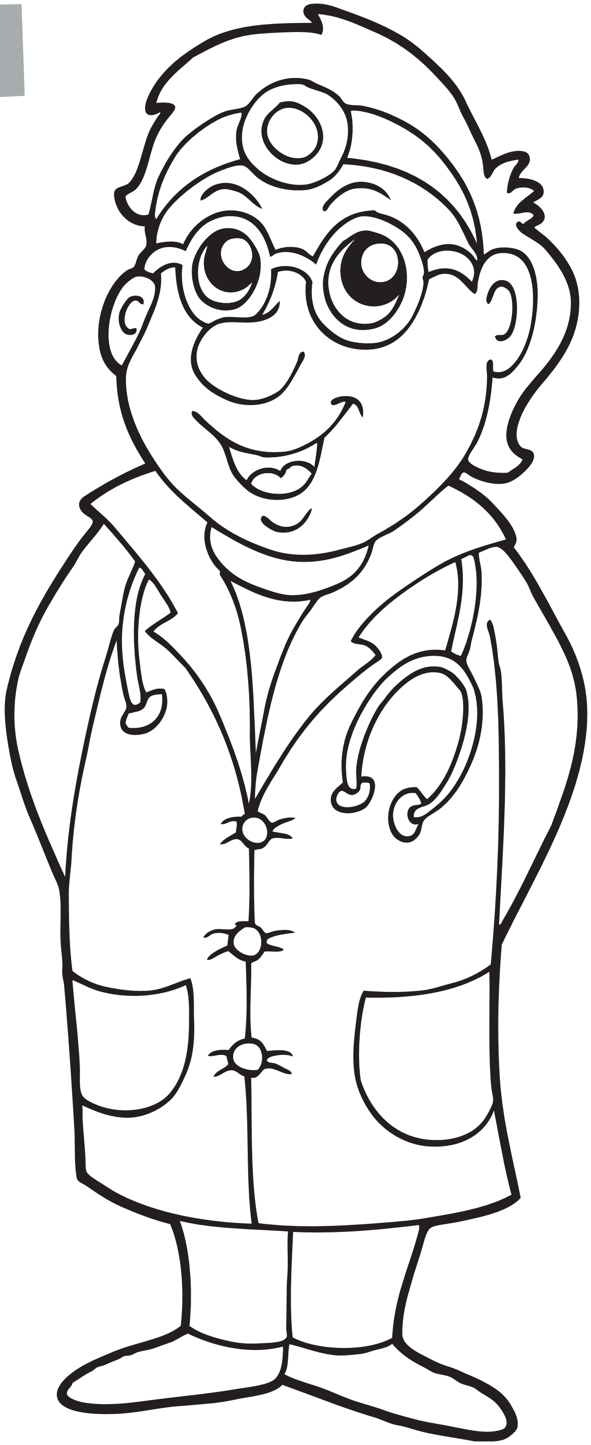

lena

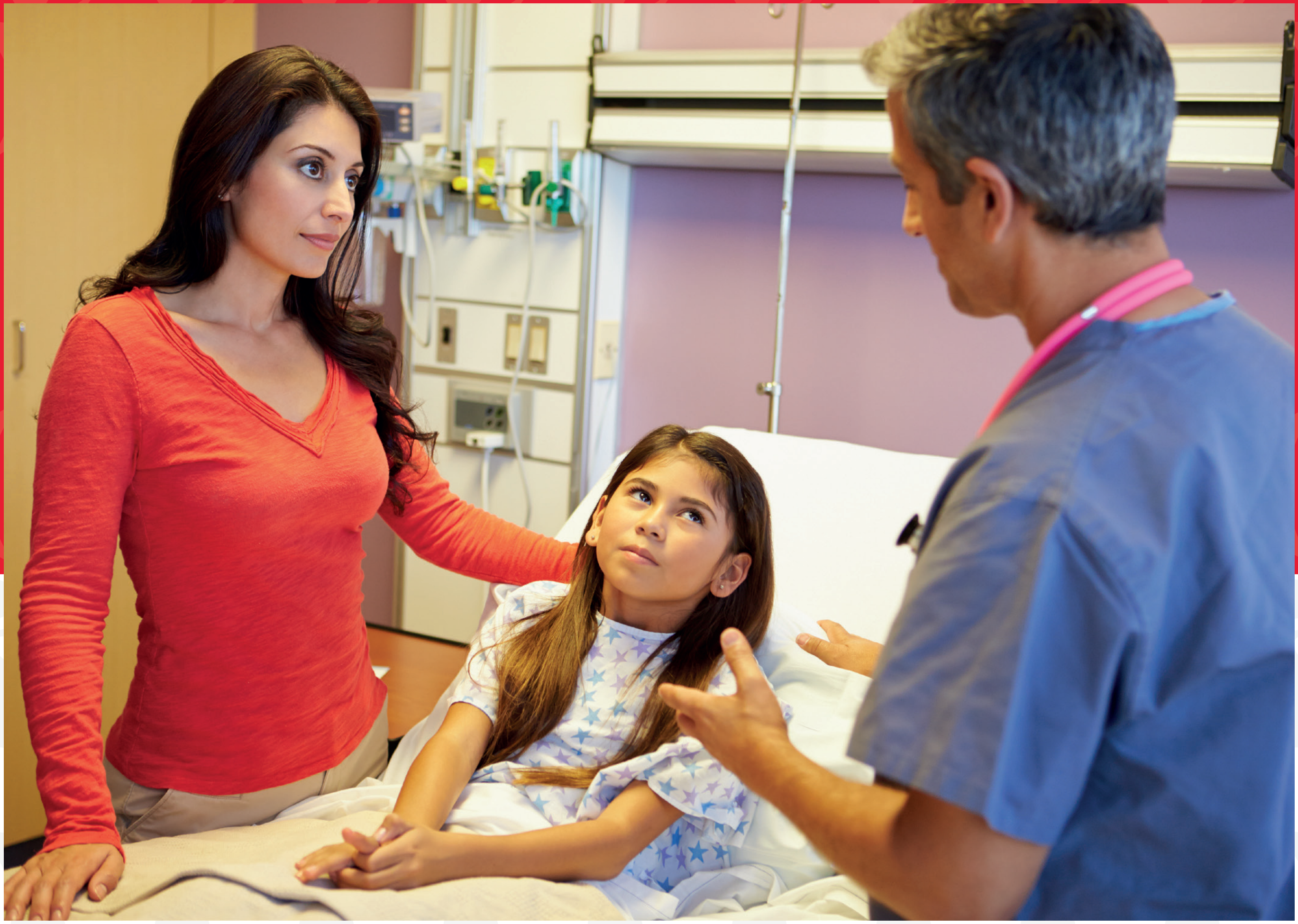

Your family will be close by

and you'll have a friendly chat with the doctor.

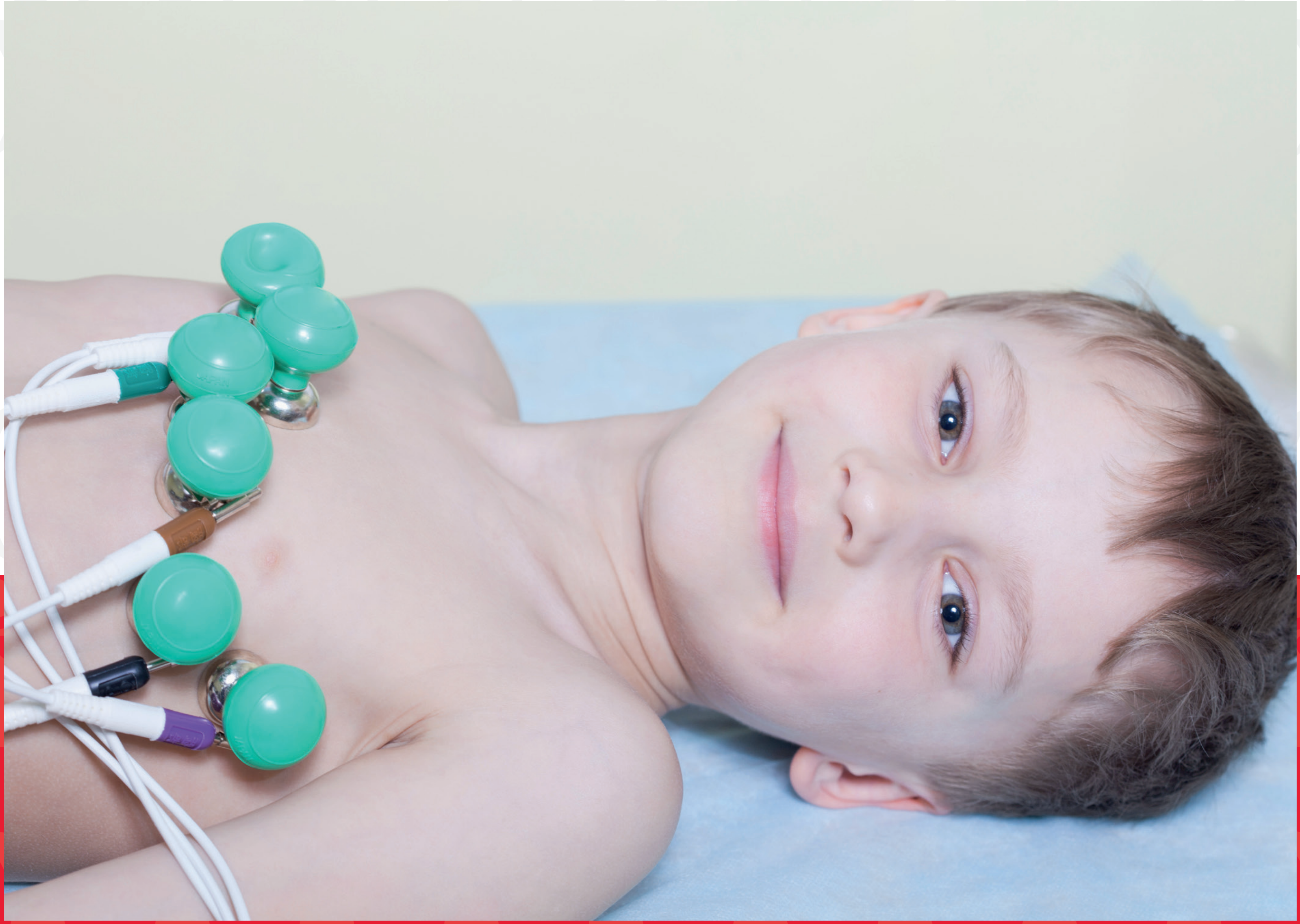

The doctor will listen to your heart [ECG]

and print out a special heart report.

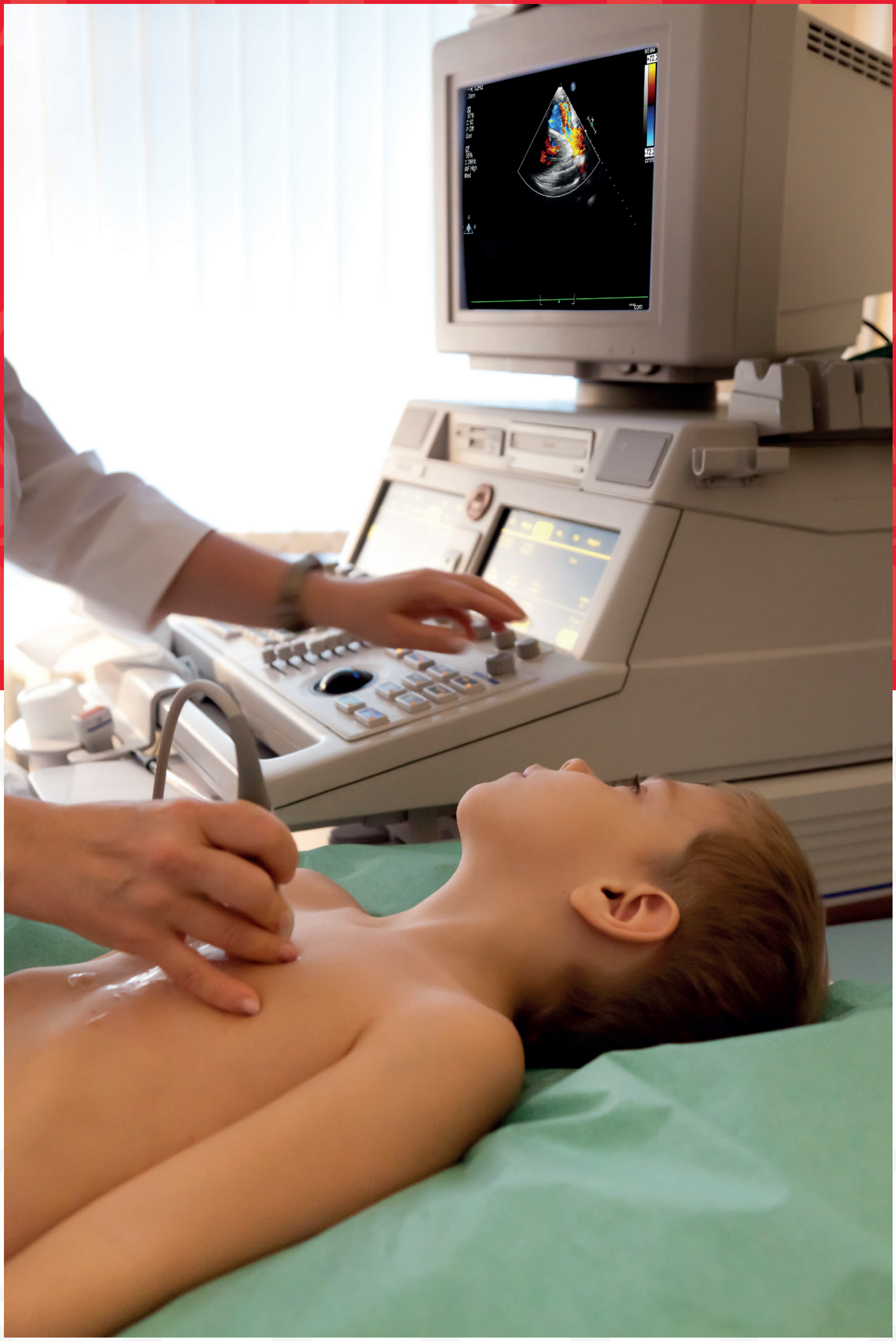

The doctor will scan your heart and look at your heartbeat.

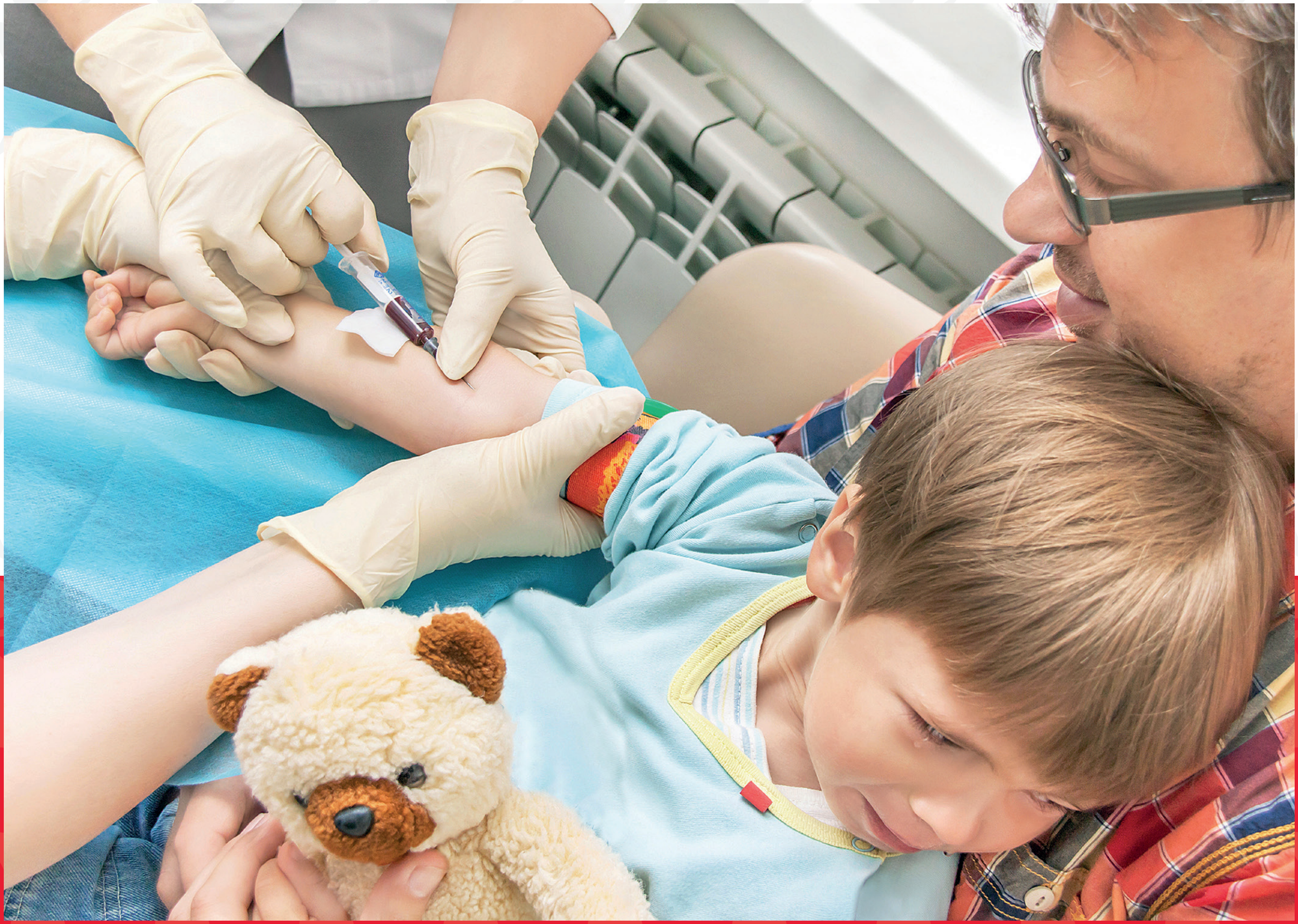

Your blood will be taken with a needle

during each visit to the hospital.

This will feel like a little scratch.

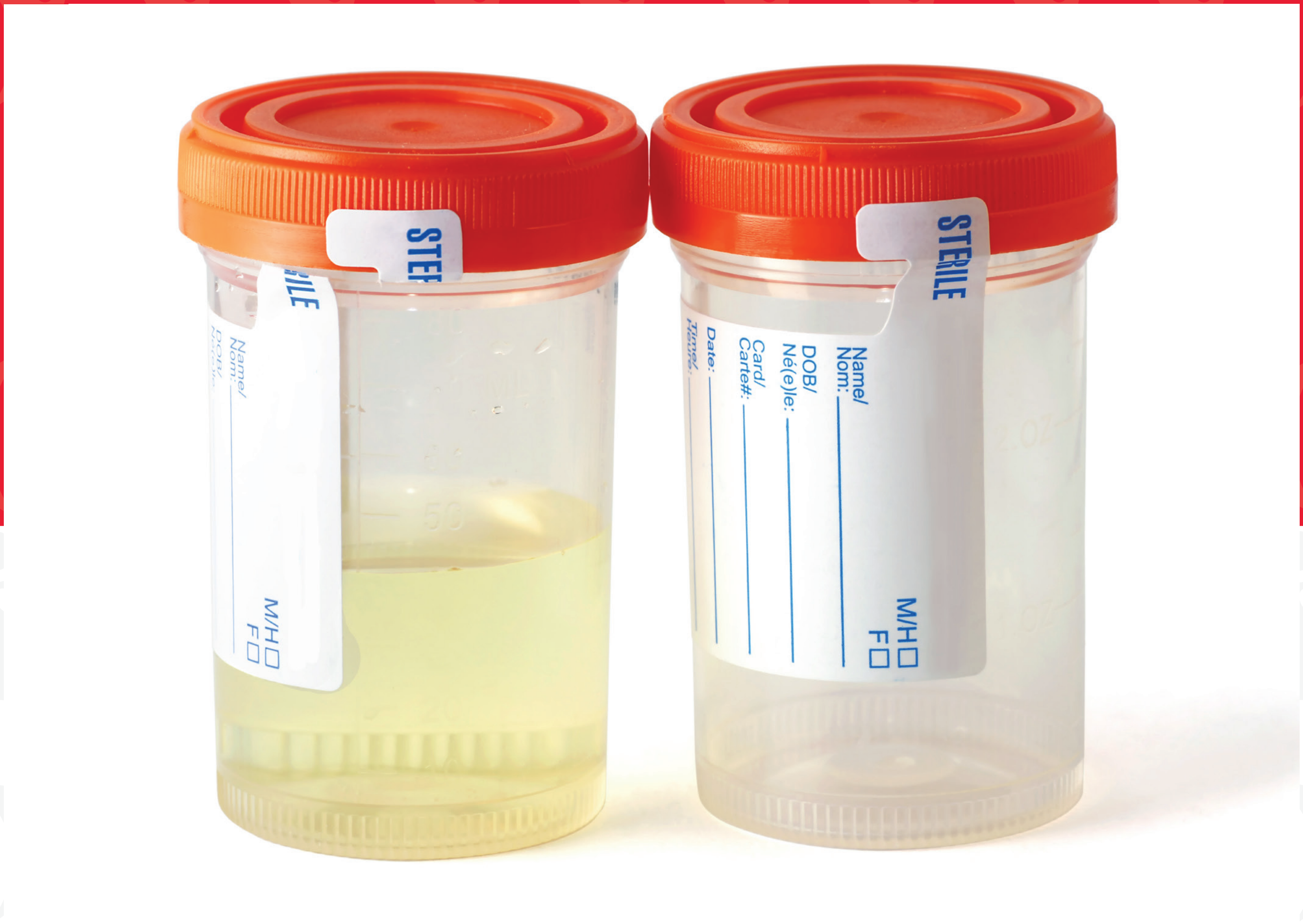

Your wee will be taken.

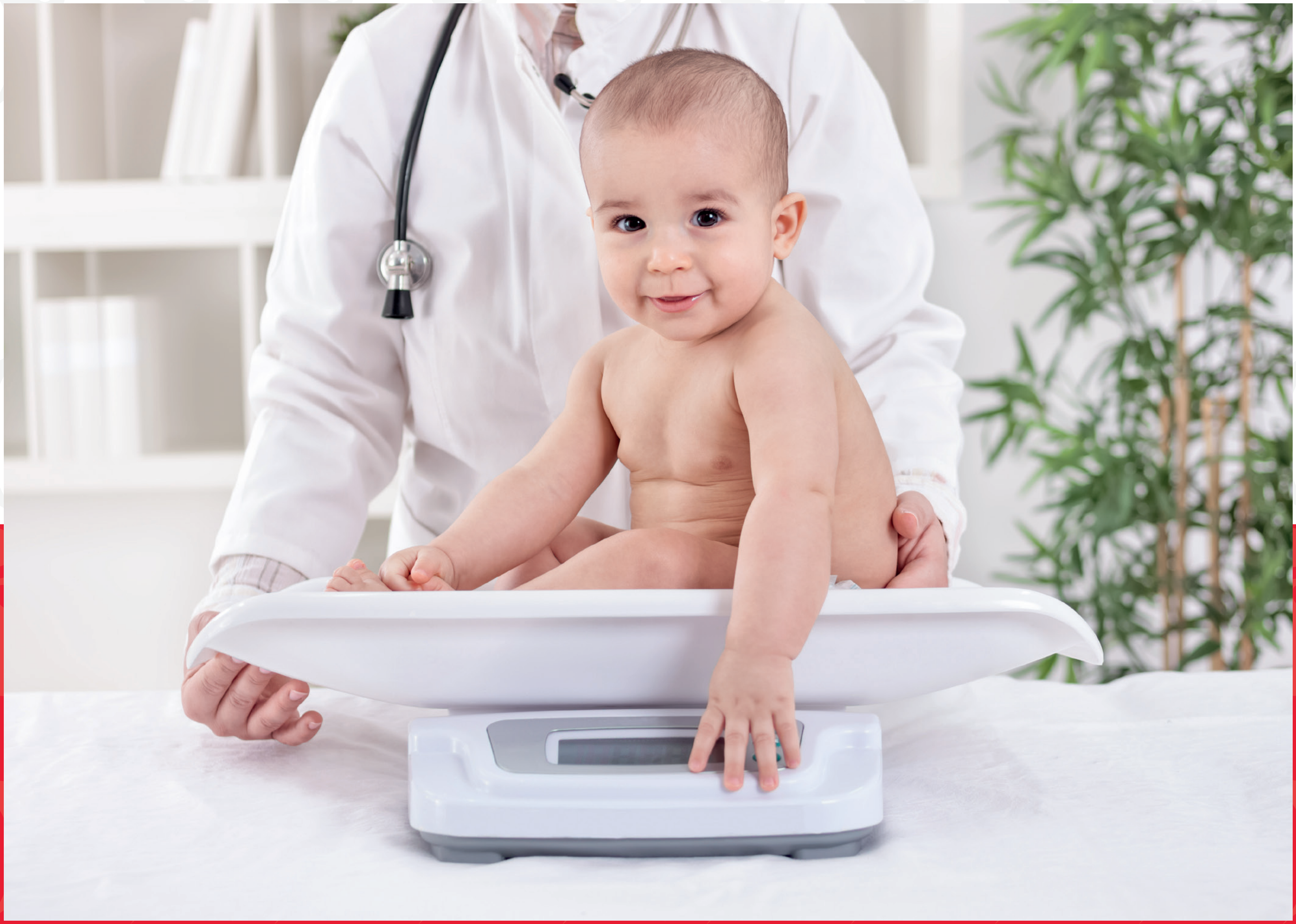

Your weight will be taken.

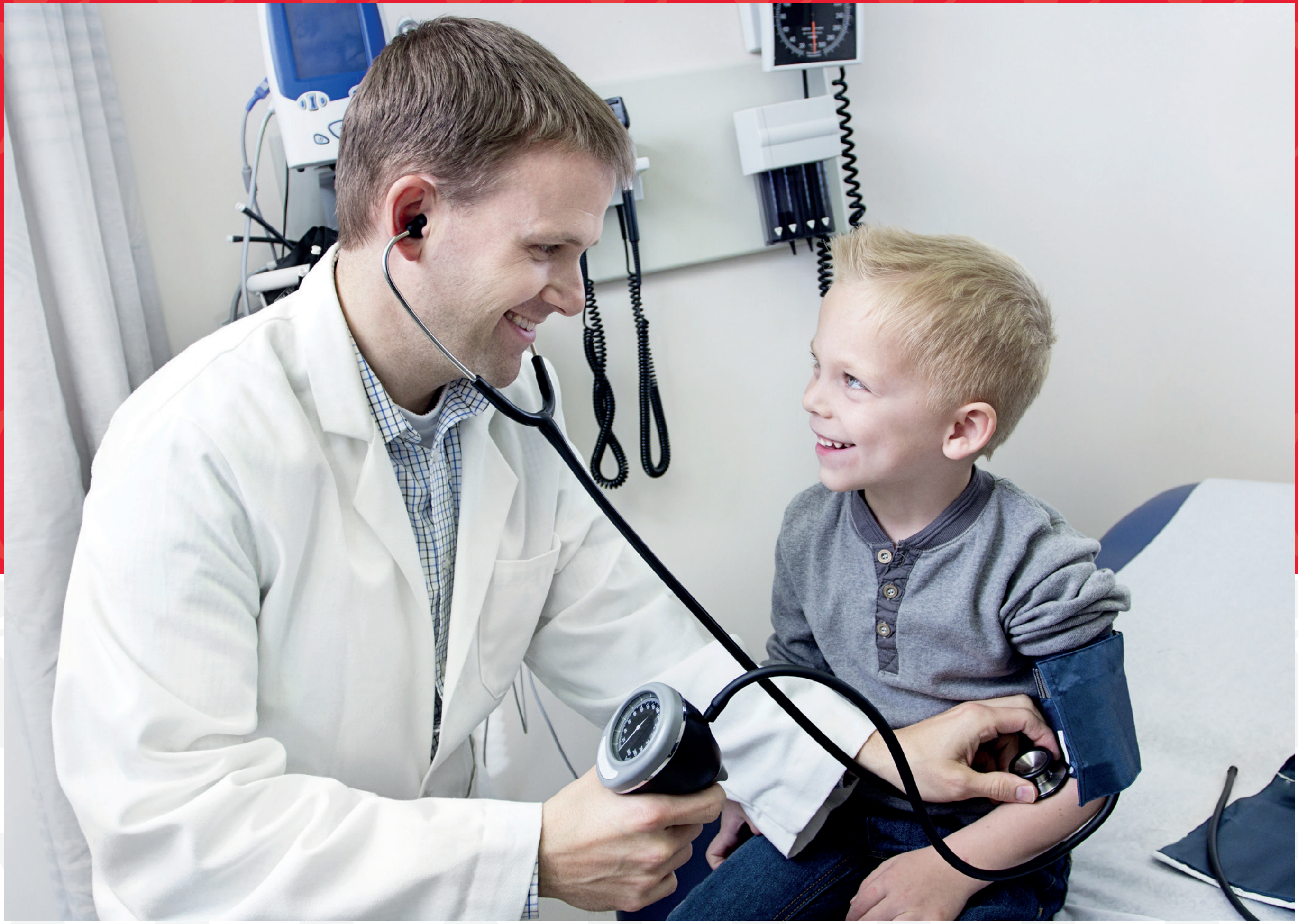

Your blood pressure will be taken

with a special monitor.

At some of your visits this will take many hours.

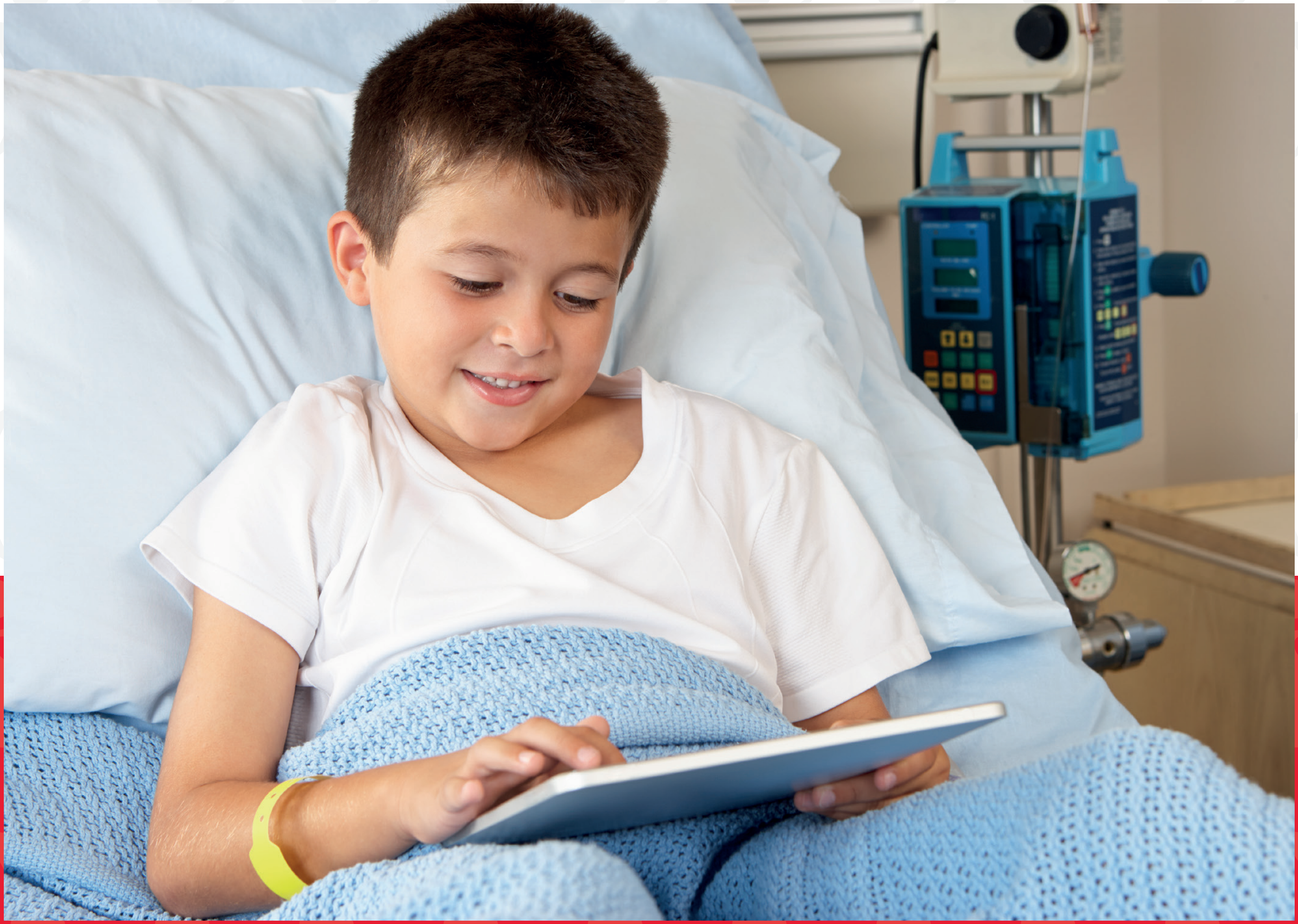

You may spend up to a whole day in hospital

so please bring your favourite things to play with.

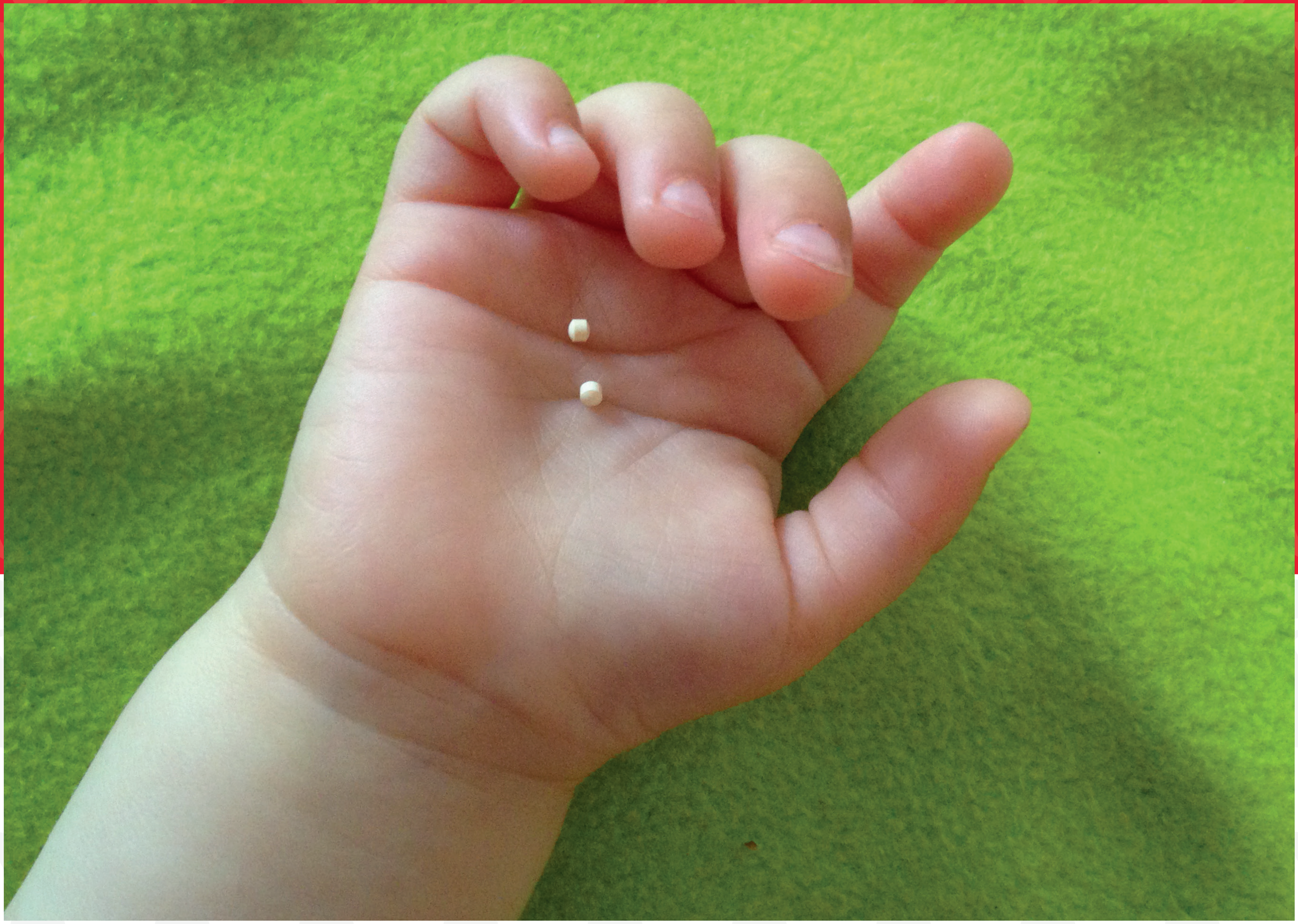

You will be asked to take mini-tablets

at the hospital during your visit.

Your mum or dad will give you mini-tablets at home.

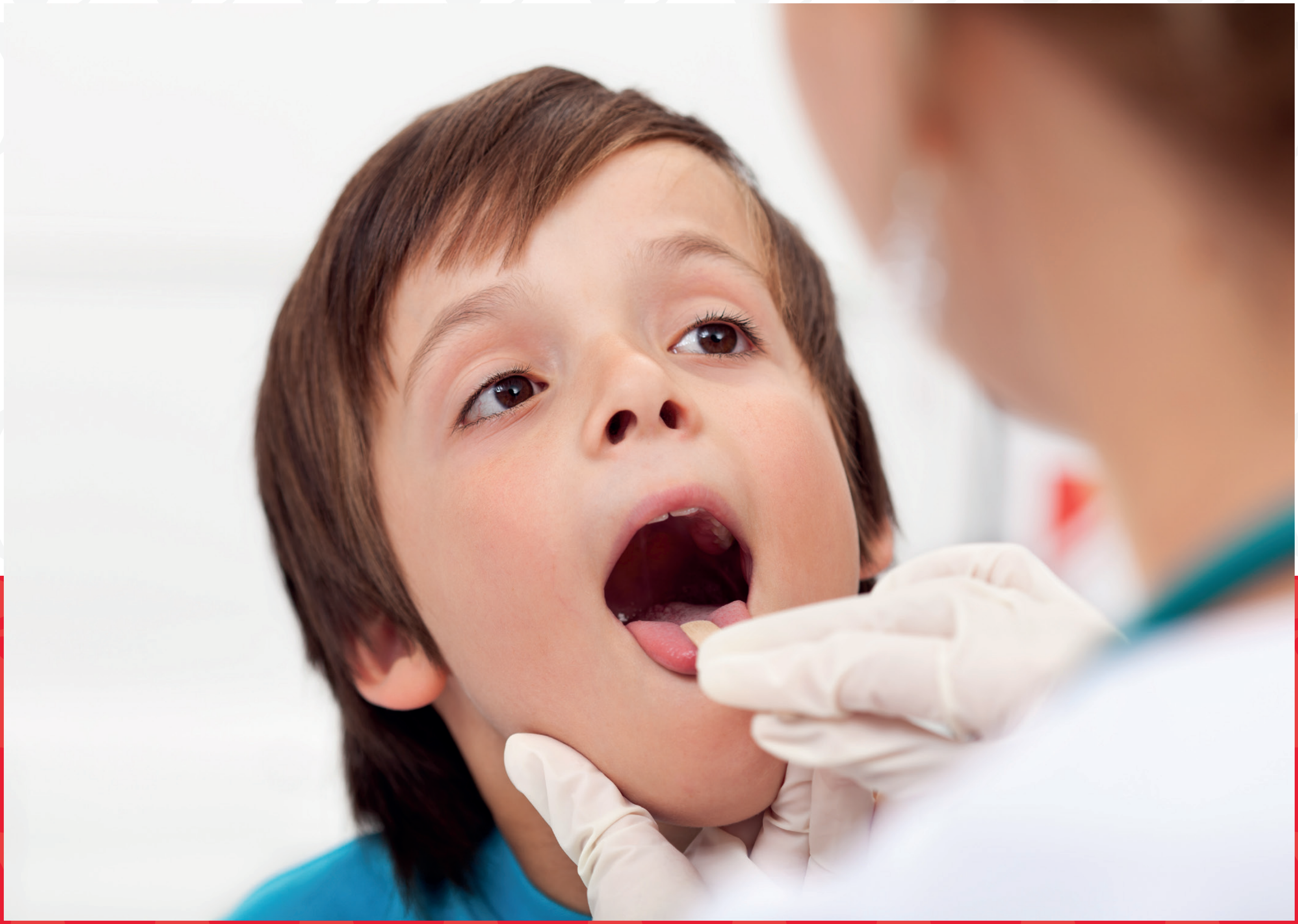

The doctor will check your mouth to see that the mini-tablets are all gone.

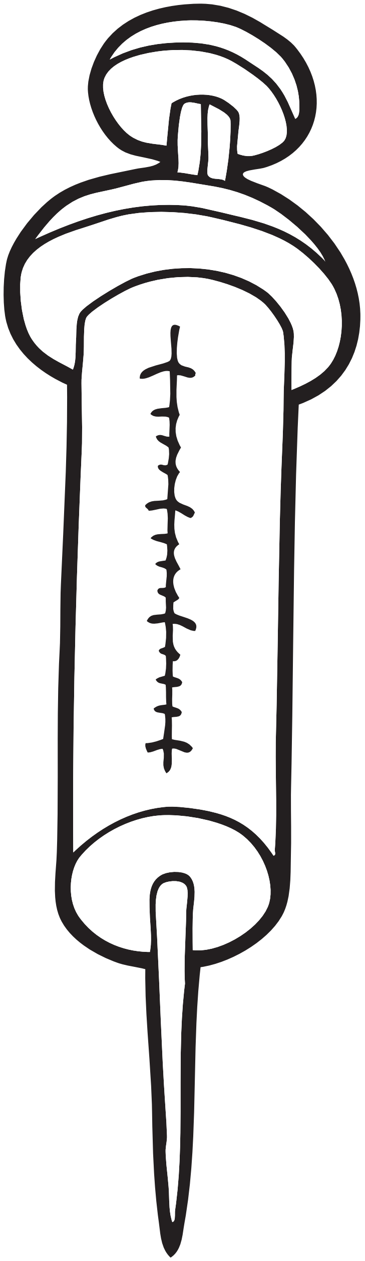

SYRINGE

STETHOSCOPE

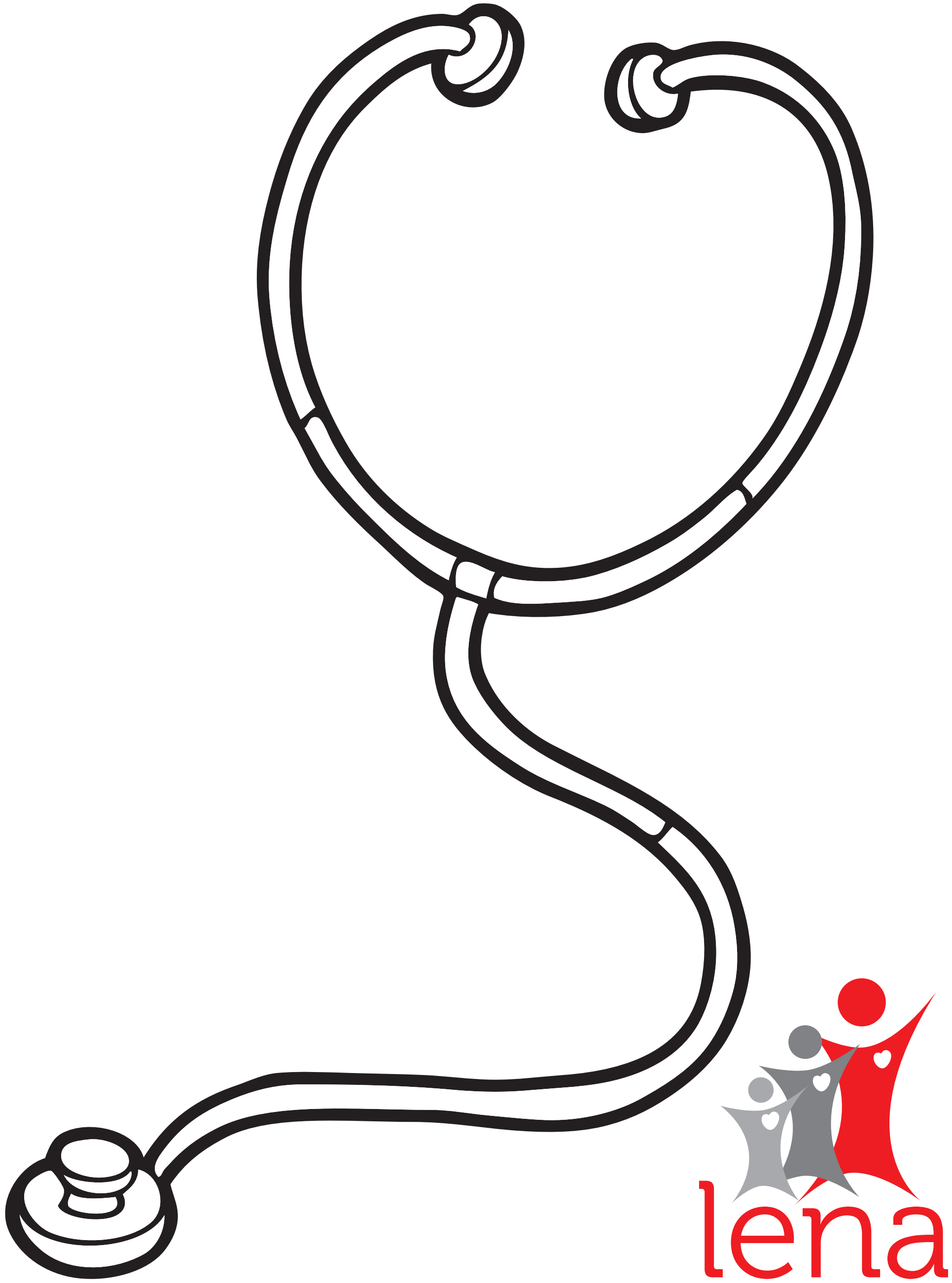

ask questions
if you're worried

?

the LENA study
is voluntary

you can choose whether
you want to take part

lena

I WANT TO TAKE PART

doctor .... date

child ....
